# Supplementary material for: Understanding drivers of family planning in rural northern India: An integrated mixed-methods approach
Source: PLoS One. 2021 Jan 13;16(1):e0243854. doi: 10.1371/journal.pone.0243854 (PMC7806122; doi:10.1371/journal.pone.0243854)
Supplement: S2 Appendix — (DOCX) [file pone.0243854.s002.docx]

**Datasets and methodology for selecting blocks for journey mapping and decision games**

**Number of blocks selected:**

1. A total of 6 blocks with 3 TSU blocks and 3 non-TSU blocks for Journey Mapping
2. A total of 24 blocks with 18 TSU blocks and 6 non-TSU blocks for decision games

**Datasets and indicators used:**

1. To select the TSU blocks, TSU’s 2015-16 Program Monitoring Community (PMC) data was used. Data were collated by TSU’s community resource persons during their monthly cluster meetings with groups of Accredited Social Health Activists (ASHAs). Data was collected to be able to compute percentages of currently pregnant women registered, women who received 3 antennal check-ups, women who delivered in a health facility, women who received any postnatal care home visits after delivery and couples currently using any modern contraceptive method.
2. To select the non-TSU blocks, Government of India’s 2015-16 Health Management Information System (HMIS) data for Uttar Pradesh was used. The data was accessed to compute percentages of currently pregnant women registered, women who received 3 antennal check-ups, women who delivered in a health facility, women who received any postnatal check-up within 48 hours of giving birth and couples currently using any modern contraceptive method.

**Block selection methodology:**

*Step 1: Classification of blocks by indicator*

First, blocks were classified as good, average and poor for each of the indicators based on the criteria specified in the table below.

Note: Different indicators have different cut off points. The distribution of each indicator governed the respective cut off points.

| Indicator (data source) | Good | Average | Poor |
| --- | --- | --- | --- |
| % currently pregnant women registered (PMC) | >75 | 55-75 | <55 |
| % women who received 3 antennal check-ups (PMC) | >70 | 50-70 | <50 |
| % women who delivered in a health facility (PMC) | >80 | 60-80 | <60 |
| % women who received any postnatal care home visits after delivery (PMC) | >90 | 65-90 | <65 |
| % couples currently using any modern contraceptive method (PMC) | >50 | 25-50 | <25 |
| % currently pregnant women registered (HMIS) | >85 | 60-85 | <60 |
| % women who received 3 antennal check-ups (HMIS) | >80 | 50-80 | <50 |
| % women who delivered in a health facility (HMIS) | >70 | 40-70 | <40 |
| % women who received any postnatal check-up within 49 hours of giving birth (HMIS) | >70 | 40-70 | <40 |
| % couples currently using any modern contraceptive method (HMIS) | >10 | 5-10 | <5 |

*Step 2: Overall classification of blocks*

Good: if 3 or more of the 5 indicators are “good”

Poor: if 3 or more of the 5 indicators are “poor”

Average: all other remaining combinations

*Step 3: Final selection of blocks*

1. **Journey Mapping**: A total of 3 TSU blocks and 3 non TSU blocks were selected. One each of “good”, “average” and “poor” blocks were selected for both types of blocks. Further, 2 of the 3 TSU blocks selected also satisfied the condition of having an active SHG program.
2. **Decision Games:** 18 TSU blocks and 6 non-TSU blocks were selected. 6 each of “good”, “average” and “poor” blocks were selected for TSU blocks and 2 each of good”, “average” and “poor” blocks were selected for non-TSU blocks. Further, 3 TSU blocks selected (1 “good”, 1 “average” and 1“poor”) also satisfied the condition of having an active SHG program.
