# Supplementary material for: Understanding drivers of family planning in rural northern India: An integrated mixed-methods approach
Source: PLoS One. 2021 Jan 13;16(1):e0243854. doi: 10.1371/journal.pone.0243854 (PMC7806122; doi:10.1371/journal.pone.0243854)
Supplement: S3 Appendix — (PDF) [file pone.0243854.s003.pdf]

In-depth immersion interview discussion guide

1. Introduction - Cover Story

We are wanting to gather all the expertise that households like yours have about healthcare, so that we can share it with other families and help them improve their wellbeing.

हम उन सभी विशेषज्ञों को एकत्रित करना चाह रहे हैं जो आप जैसे घरों में हेल्थकेयर के बारे में हैं, जिससे कि हम इसे अन्य परिवारों को शेयर कर सकते और उनकी तंदरूस्तीमें सुधार के लिए उन्हें मदद मिल सके।

2. Household background

- Who is living in the household?
    - घर में कौन रह रहा है?
- How many grandchildren live in the household?
    - घर में कितने पोता/पोती रहते हैं?
- How long have you been married?
    - आपकी शादी को कितने वर्ष हो गए हैं?
- How many children do you have? How old are they?
    - आपके कितने बच्चे हैं? वे कितने वर्ष के हैं?
- To what grade did you study? (ask for mother, husband,MIL)
- आपने किस ग्रेड तक पढ़ाई की थी? (माँ, पति, सास के लिए पूछें)
- If pregnant, how far along are you?(month)
    - यदि गर्भवती हो, तो आप कितने महीने की गर्भवती हैं?

3. Health beliefs open ended

What do you think are the most important things to do for the health of your baby:
आपके विचार में आपके बच्चे के स्वास्थ्य के लिए करने के लिए सबसे महत्वपूर्ण चीजें क्या हैं :

● During pregnancy? Why? How do you think it benefits thebaby?
गर्भावस्था के दौरान? क्यों? आपके विचार में ये बच्चे को कैसे फायदा पहुँचाता है?

● At time of delivery / birth? Why? How do you think it benefits thebaby?
डिलीवरी/जन्म के समय? क्यों? आपके विचार में ये बच्चे को कैसे फायदा पहुँचाता है?

● After birth (first month)? Why? How do you think it benefits thebaby?
जन्म के बाद (पहले महीने)? क्यों? आपके विचार में ये बच्चे को कैसे फायदा पहुँचाता है?

What do you think are the most important things to do for the health of the mother:
आपके विचार में माँ के स्वास्थ्य के लिए करने के लिए सबसे महत्वपूर्ण चीजें क्या हैं :

● During pregnancy? Why? How do you think it benefits themother?
गर्भावस्था के दौरान? क्यों? आपके विचार में ये माँ को कैसे फायदा पहुँचाता है?

● At time of delivery / birth? Why? How do you think it benefits the mother?

● डिलीवरी/जन्म के समय? क्यों? आपके विचार में ये माँ को कैसे फायदा पहुँचाता है?

● After birth (first month)? Why? How do you think it benefits the mother?
जन्म के बाद (पहले महीने)? क्यों? आपके विचार में ये माँ को कैसे फायदा पहुँचाता है?

4. FAMILY PLANNING

A. Aspirations

How many children / sexes do you want? Why?

आप कितने बच्चे/सेक्स को करना चाहती हैं? क्यों?

What do you think is the ideal spacing of time between marriage and the first child? Between subsequent children? Why?

आपके विचार में शादी और पहले बच्चे के बीच में आदर्श समय का अंतराल क्या होता है? बाद के बच्चों के बीच में? क्यों?

B. Efficacy

Do you / did you feel you are in control these decisions? If not, who or what combination of people are? (husband, MIL, other?) Why do you think they are in control of the decision? Why do you feel you need to follow their decision? How would you feel if they weren’t in control of the decision? Would you make a different decision if they weren’t in control?

क्या आप महसूस करती हैं/क्या आपने महसूस किय ाकि आप इन निर्णयों के नियंत्रण में है? यदि नहीं, तो कौन या

लोगों का कौन-सा कम्बाईनेशन है? (पति, सास, अन्य?) आपके विचार में वे निर्णय के नियंत्रण में क्यों हैं? आप क्यों महसूस करती हैं कि आपको उनके निर्णय का अनुसरण करने की जरूरत है? यदि वे निर्णय के नियंत्रण में न हों तो आप कैसा महसूस करेंगी? यदि वे नियंत्रण में न हों तो क्या आप अलग निर्णय लेंगी?

C. **Use of FP**

Are you using any method to control when you have children? If yes, did you use before your first child? After which child? What method? Why that method? What do you most like about that method (ease of using, lack of side effects, etc)? What do you least like about that method (difficulty in using, side effects,etc)?

क्या आप बच्चे न हों इसके नियंत्रण के लिए कोई तरीके का इस्तेमाल कर रही हैं? यदि हाँ, तो क्या आपने अपने पहले बच्चे से पहले इस्तेमाल किया था? किस बच्चे के बाद? किस तरीके को? वह तरीका ही क्यों? आप उस तरीके (इस्तेमाल करने की आसानी, साइड एफेक्ट्स की कमी आदि) के बारे क्या सबसे ज्यादा पसंद करती है।?आप उस तरीके (इस्तेमाल करने में मुश्किल, साइड एफेक्ट्स आदि) के बारे में सबसे कम क्या पसंद करती हैं?

D. Influence

Did your mother use a family planning method? Which? How was that experience? Do you know of any friends that use a family planning method? Which? How was that experience? Do you remember talking about family planning with anyone? If so, who? Your husband? Your mother-in-law? The ASHA? A nurse? A combination of people?
OfcoursetheASHA/providerisnotadoctor-doyoutrustherinputandinformation?
Why / why not?

क्या आपकी माँ ने परिवार नियोजन के तरीके का इस्तेमाल किया था? किसका? उनका अनुभव कैसा था? क्या आप किसी ऐसे दोस्त को जानती हैं, जो परिवार नियोजन के तरीके का इस्तेमाल करती हैं? किसका? उनका अनुभव कैसा था? क्या आपको किसी से परिवार नियोजन के बारे में बात करना याद है? यदि ऐसा है, तो किससे? अपने पति? अपनी सास? आशा? नर्स? लोगों के कम्बाईनेशन? वास्तव में आशा/प्रोवाइडर डॉक्टर नहीं हाते, क्या आप उनके इनपुट और जानकारी पर भरोसा करती हैं? क्यों? क्यों नहीं?

If YES:

यदि हाँ तो :

Do you remember when this discussion happened approximately? Were you just married, were you pregnant? With first, second, third child? Was it after giving birth? To first, second, third child? (try to find out when in the context of their life)

क्या आपको याद है कि यह डिस्कशन लगभग कब हुआ था? जैसे ही आपकी शादी हुई थी, क्या जब आप गर्भवती थी? पहले, दूसरे, तीसरे बच्चे के साथ? क्या यह जन्म देने के बाद था? पहले, दूसरे, तीसरे बच्चे के लिए? (उनके जीवन के संदर्भ में यह जानने की कोशिश करें)

How did the conversation start / what prompted it?

बातचीत कैसे शुरू हुई थी? इसके लिए किसने प्रेरित किया था?

Where do you remember the conversation taking place? At home? At a hospital? At a community event?

क्या आपको याद है कि बातचीत कहाँ पर हुई थी? घर में? अस्पताल में? कम्युनिटी/सादायिक इवेंट में?

What do you remember being discussed? Who expressed opinions on what to do? What were those opinions? What information was shared?

आपको हुए डिस्कश के बारे में क्या याद है? क्या करना है इस पर किसने राय व्यक्त की? उनकी राय क्या थी? कौन-सी जानकारी शेयर की गयी थी?

In that conversation, who most influenced your thinking? Why?

उस बातचीत में आपके विचार से कौन सबसे ज्यादा प्रभावित हुआ? क्यों?

What was the result of the conversation? Did you form an opinion on whether Family Planning was right for you? Did you make a decision on whether to pursue family planning? If so what method? Why that method?

बातचीत का परिणाम क्या था? क्या आपने इस बात पर एक राय बनायी थी कि परिवार नियोजन आपके लिए सही था? क्या आपने परिवार नियोजन को आगे बढ़ाने के लिए कोई निर्णय लिया था? यदि ऐसा है तो किस तरीके को? वह तरीका ही क्यों?

WOULD YOU CHOOSE THAT METHOD AGAIN IN THE FUTURE? WHY OR WHY NOT?

क्या आप भविष्य में फिर से उस तरीके को चुनेंगी? क्यों या क्यों नहीं?

Beliefs
How do you think family planning benefits you? Your family?
आपके विचार में परिवार नियोजन आपको कैसे फायदा पहुँचाता है? आपके परिवार को?
Did you discuss Copper-T?
क्या आपने कॉपर टी पर डिस्कश किया था?
If so, what do you like most about this option (easy to use, easy to access, no side effects)? What do you least like about this option (difficult to use, difficult to get, side effects - what)? Do you expect to get paid for this procedure? If so, how much?
यदि ऐसा है, तो आप इस राय (इस्तेमाल में आसान, एक्सेस करने में आसान, कोई साइड एफेक्ट्स नहीं) के बारे में सबसे ज्यादा क्या पसंद करती हैं? आप इस राय (इस्तेमाल में मुश्किल, लेने में मुश्किल, कौन-सा साइड एफेक्ट्स) के बारे में क्या सबसे कम पसंद करती हैं? क्या आप इस तरीके के लिए भुगतान करने की अपेक्षा करती हैं? यदि ऐसा है तो कितना?

Did you discuss IUCD?
क्या आपने आईयूसीडी पर डिस्कश किया था?
If so, what do you like most about this option (easy to use, easy to access, no side effects)? What do you least like about this option (difficult to use, difficult to get, side effects - what)? Do you expect to get paid for this procedure? If so, howmuch?
यदि ऐसा है, तो आप इस राय (इस्तेमाल में आसान, एक्सेस करने में आसान, कोई साइड एफेक्ट्स नहीं) के बारे में सबसे ज्यादा क्या पसंद करती हैं? आप इस राय (इस्तेमाल में मुश्किल, लेने में मुश्किल, कौन-सा साइड एफेक्ट्स) के बारे में क्या सबसे कम पसंद करती हैं? क्या आप इस तरीके के लिए भुगतान करने की अपेक्षा करती हैं? यदि ऐसा है तो कितना?

Did you discuss sterilization?
क्या आपने नसबंदी पर डिस्कश किया था?

If so, what do you like most about this option (easy to use, easy to access, no side effects)? What do you least like about this option (difficult to use, difficult to get, side effects - what)? Do you expect to get paid for this procedure? If so, howmuch?

यदि ऐसा है, तो आप इस राय (इस्तेमाल में आसान, एक्सेस करने में आसान, कोई साइड एफेक्ट्स नहीं) के बारे में सबसे ज्यादा क्या पसंद करती हैं? आप इस राय (इस्तेमाल में मुश्किल, लेने में मुश्किल, कौन-सा साइड एफेक्ट्स) के बारे में क्या सबसे कम पसंद करती हैं? क्या आप इस तरीके के लिए भुगतान करने की अपेक्षा करती हैं? यदि ऐसा है तो कितना?

Did you discuss condoms?
क्या आपने कंडोम पर डिस्कश किया था?

If so, what do you like most about this option (easy to use, easy to access, no side effects)? What do you least like about this option (difficult to use, difficult to get, side effects - what)? Do you expect to get paid for this procedure? If so, howmuch?
यदि ऐसा है, तो आप इस राय (इस्तेमाल में आसान, एक्सेस करने में आसान, कोई साइड एफेक्ट्स नहीं) के बारे में सबसे ज्यादा क्या पसंद करती हैं? आप इस राय (इस्तेमाल में मुश्किल, लेने में मुश्किल, कौन-सा साइड एफेक्ट्स) के बारे में क्या सबसे कम पसंद करती हैं? क्या आप इस तरीके के लिए भुगतान करने की अपेक्षा करती हैं? यदि ऐसा है तो कितना?

Did you discuss injections?
क्या आपने इंजेक्शन पर डिस्कश किया था?
If so, what do you like most about this option (easy to use, easy to access, no side effects)? What do you least like about this option (difficult to use, difficult to get, side effects - what)? Do you expect to get paid for this procedure? If so, howmuch?
यदि ऐसा है, तो आप इस राय (इस्तेमाल में आसान, एक्सेस करने में आसान, कोई साइड एफेक्ट्स नहीं) के बारे में सबसे ज्यादा क्या पसंद करती हैं? आप इस राय (इस्तेमाल में मुश्किल, लेने में मुश्किल, कौन-सा साइड एफेक्ट्स) के बारे में क्या सबसे कम पसंद करती हैं? क्या आप इस तरीके के लिए भुगतान करने की अपेक्षा करती हैं? यदि ऐसा है तो कितना?

Did you discuss pills?
क्या आपने गोलियों पर डिस्कश किया था?
If so, what do you like most about this option (easy to use, easy to access, no side effects)? What do you least like about this option (difficult to use, difficult to get, side effects - what)? Do you expect to get paid for this procedure? If so, howmuch?
यदि ऐसा है, तो आप इस राय (इस्तेमाल में आसान, एक्सेस करने में आसान, कोई साइड एफेक्ट्स नहीं) के बारे में सबसे ज्यादा क्या पसंद करती हैं? आप इस राय (इस्तेमाल में मुश्किल, लेने में मुश्किल, कौन-सा साइड एफेक्ट्स) के बारे में क्या सबसे कम पसंद करती हैं? क्या आप इस तरीके के लिए भुगतान करने की अपेक्षा करती हैं? यदि ऐसा है तो कितना?

If NO:

यदि नहीं, तो :
Influence
Do you remember any time in which you thought about how to control when to have children? If so, when? Were you just married, were you pregnant? Was it after giving birth? (try to find out when in the context of their life)
क्या आपको किसी ऐसे समय के बारे में याद हैं, जिसमें आपने इस बारे में सोचा कि बच्चे न हों इस पर कैसे नियंत्रण किया जाए? यदि ऐसा है तो कब? ? जैसे ही आपकी शादी हुई थी, क्या जब आप गर्भवती थी? क्या यह जन्म देने के बाद था?(उनके जीवन के संदर्भ में यह जानने की कोशिश करें)

Where do you remember being at when you had these thoughts? At home? At a hospital? At a community event?
आपको उस समय कहाँ पर होना याद है, जब आपके पास ये विचार थे? घर में? अस्पताल में? कम्युनिटी/सामुदायिक इवेंट में?

What do you remember thinking about? What opinions did you have? What were those opinions? Did you feel a desire to seek out any information or other people’s thoughts? If so, what were you hoping to learn or understand?
आपको इसके बारे में क्या सोचना याद है? आपकी क्या राय थी? उनकी राय क्या थी? क्या आपको किसी जानकारी को लेने या अन्य लोगों के विचारों को लेने की इच्छा हुई? यदि ऐसा है तो, आप क्या सीखने या समझने की उम्मीद कर रही थी?

What was the result of your thoughts? Did you take any action? Talk to anyone? Decide whether to pursue family planning? If so what method? Why that method?
आपके विचारों का परिणाम क्या था? क्या आपने कोई कदम उठाया था? किसी से बात किया था? क्या परिवार नियोजन को आगे बढ़ाने का निर्णय लिया थ? यदि ऐसा है तो किस तरीके को? वह ही तरीका क्यों?

What influenced your decision most? (who or what) Why?
आपके निर्णय से कौन सबसे ज्यादा प्रभावित हुआ था? (कौन या क्या) क्यों?

WOULD YOUR DECISION BE THE SAME OR DIFFERENT IF YOU MADE IT AGAIN TODAY? WHY?

यदि आप आज फिर से इस पर निर्णय लें तो क्या आपका निर्णय वही होगा या अलग होगा? क्यों?

Beliefs

We would like to know what you think about a few family planning methods:
हम जानना चाहेंगे कि आप परिवार नियोजन के तरीकों के बारे में क्या सोचती हैं :

Sterilization
नसबंदी

What do you (would) like most about this option (easy to use, easy to access, no side effects)? What do (would) you least like about this option (difficult to use, difficult to get, side effects - what)? Do you expect to get paid for this procedure? If so, how much?
आप इस राय (इस्तेमाल में आसान, एक्सेस करने में आसान, कोई साइड एफेक्ट्स नहीं) के बारे में सबसे ज्यादा क्या पसंद करती हैं? /क्या पसंद करेंगी? आप इस राय (इस्तेमाल में मुश्किल, लेने में मुश्किल, कौन-सा साइड एफेक्ट्स) के बारे में क्या सबसे कम पसंद करती हैं? /क्या पसंद करेंगी? क्या आप इस तरीके के लिए भुगतान करने की अपेक्षा

करती हैं? यदि ऐसा है तो कितना?

Condoms
कंडोम

What do you (would) like most about this option (easy to use, easy to access, no side effects)? What do (would) you least like about this option (difficult to use, difficult to get, side effects - what)? Do you expect to get paid for this procedure? If so, how much?
आप इस राय (इस्तेमाल में आसान, एक्सेस करने में आसान, कोई साइड एफेक्ट्स नहीं) के बारे में सबसे ज्यादा क्या पसंद करती हैं? /क्या पसंद करेंगी? आप इस राय (इस्तेमाल में मुश्किल, लेने में मुश्किल, कौन-सा साइड एफेक्ट्स) के बारे में क्या सबसे कम पसंद करती हैं? /क्या पसंद करेंगी? क्या आप इस तरीके के लिए भुगतान करने की अपेक्षा करती हैं? यदि ऐसा है तो कितना?

Injections
इंजेक्शन

What do you (would) like most about this option (easy to use, easy to access, no side effects)? What do (would) you least like about this option (difficult to use, difficult to get, side effects - what)? Do you expect to get paid for this procedure? If so, how much?
आप इस राय (इस्तेमाल में आसान, एक्सेस करने में आसान, कोई साइड एफेक्ट्स नहीं) के बारे में सबसे ज्यादा क्या पसंद करती हैं? /क्या पसंद करेंगी? आप इस राय (इस्तेमाल में मुश्किल, लेने में मुश्किल, कौन-सा साइड एफेक्ट्स) के बारे में क्या सबसे कम पसंद करती हैं? /क्या पसंद करेंगी? क्या आप इस तरीके के लिए भुगतान करने की अपेक्षा करती हैं? यदि ऐसा है तो कितना?

Pills
गोलियां

What do you (would) like most about this option (easy to use, easy to access, no side effects)? What do (would) you least like about this option (difficult to use, difficult to get, side effects - what)? Do you expect to get paid for this procedure? If so, how much?
आप इस राय (इस्तेमाल में आसान, एक्सेस करने में आसान, कोई साइड एफेक्ट्स नहीं) के बारे में सबसे ज्यादा क्या पसंद करती हैं? /क्या पसंद करेंगी? आप इस राय (इस्तेमाल में मुश्किल, लेने में मुश्किल, कौन-सा साइड एफेक्ट्स) के बारे में क्या सबसे कम पसंद करती हैं? /क्या पसंद करेंगी? क्या आप इस तरीके के लिए भुगतान करने की अपेक्षा करती हैं? यदि ऐसा है तो कितना?
