# Supplementary material for: Understanding drivers of family planning in rural northern India: An integrated mixed-methods approach
Source: PLoS One. 2021 Jan 13;16(1):e0243854. doi: 10.1371/journal.pone.0243854 (PMC7806122; doi:10.1371/journal.pone.0243854)
Supplement: S4 Appendix — (PDF) [file pone.0243854.s004.pdf]

HOUSEHOLD: WHEN TO HAVE CHILDREN
परिवार: बच्चे कब करने हैं

1. PRE-INTERVIEW
इंटरव्यू से पहले

- Did or did not use a famiy planning method (based on recruiter information)
  परिवार नियोजन विधि इस्तेमाल की थी या नहीं की थी (रिक्रूटर की सूचना के आधार पर)
- Name
  नाम
- Date and time
  तारीख और समय
- Age
  आयु
- Hindu or Muslim?
  हिंदू या मुसलमान?
- Caste
  जाति
- Years of schooling
  कितनेवर्ष स्कूल गई हैं?
- Block
  ब्लाक

2. INTRODUCTION
परिचय

We are conducting research to help improve the health and wellbeing of women and their babies in Uttar Pradesh. We would like to get your thoughts and opinions about how households in your community **decide and controlwhen to have children**. The interview should last about 90 minutes. Do you have any questions for me?
हम उत्तर प्रदेश में महिलाओं और उनके बच्चों के स्वास्थ्य और कल्याण में सुधार लाने में मदद करने के लिए शोधकार्य कर रहे हैं। हम इस बारे में आपके विचार और राय जानना चाहेंगे कि **आपके समुदाय के लोग कैसे यह निर्णय लेते और नियंत्रण करते हैं कि बच्चे कब करने हैं।** यह बातचीत लगभग 90 मिनट चलेगी। क्या आपके मन में मेरे लिए कोई सवाल है?

Is it OK with you that we record our discussion?
अगर हम अपनी बातचीत को रिकॉर्ड करें तो क्या यह आपके लिए ठीक रहेगा?

It for us only and just so that we can refer back to anything if our notes are unclear. We will erase the recordings shortly afterwards.

यह रिकॉर्डिंग केवल हमारे लिए है और इसलिए है कि अगर हमारे नोट्स स्पष्ट नहीं हों तो हम इसे दुबारा सुन सकें। हम उसके बाद जल्दी ही रिकॉर्डिंग को मिटा देंगे।

3. DEMOGRAPHICS
जनसांख्यिकी

Before we get started I would like to gather some basic information about your household.
इससे पहले कि हम शुरुआत करें, मैं आपके परिवार के बारे में कुछ बुनियादी जानकारी लेना चाहूंगी।

- Identify respondent (wife, husband, MIL / Matriarch, FIL / Patriarch)
  प्रत्युत्तरदाता की पहचान करें (पत्नी, पति, सास/बूढ़ी, ससुर/बूढ़ा)
- Age of respondent?
  प्रत्युत्तरदाता की उम्र?
- Religion?
  धर्म?
- Caste?
  जाति?
- Who is living in the household?
  उनके परिवार में कौन रहता है?
- Years of schooling? Did you go to high school?
  कितने साल स्कूल गई हैं? क्या आप हाई स्कूल गई हैं?
- Do you have an ASHA?
  क्या आपके पास एक ASHA है?
  - If so, when was the last time the ASHA visited the household?
    यदि हां तो पिछली बार ASHA ने परिवार से कब मुलाकात की थी?
- How long has the couple been married?
  जोड़े की शादी हुए कितना समय हो गया है?
- How many children does the couple have?
  जोड़े के कितने बच्चे हैं?
  - If any, what are their ages?
    अगर बच्चे हैं तो उनकी उम्र क्या है?
  - If any, where were they delivered at?
    अगर बच्चे हैं तो वे कहां पैदा हुए थे?
- If the couple is pregnant, how many months along?
  यदि पत्नी गर्भवती है तो कितने महीने से?
  - Had you registered the pregnancy? At what time?
    क्या आपने गर्भावस्था का पंजीकरण करवाया था? किस समय पर?

4. JOURNEY CONTEXT
यात्रा का परिप्रेक्ष्य

Because we are focused on the health of mothers and their babies, I'd like to get your thoughts on a **healthy pregnancy**.

- How do you know when the expectant mother and baby are healthy? (during pregnancy)
  आपको कैसे पता चलता है कि गर्भवती महिला और शिशु स्वस्थ हैं? (गर्भावस्था के दौरान)
    - What do you look for or expect to see?
      आप किन चीजों को देखती हैं या देखने की उम्मीद करती हैं?
- What do you think are important things to do to ensure a healthy pregnancy?
  एक स्वस्थ गर्भावस्था सुनिश्चित करने के लिए, आपके ख्याल से कौन सी चीजें जरूरी हैं?
    - Healthcare
      स्वास्थ्य देखभाल
    - Diet / nutrition
      आहार/पोषण
    - Family life within the household
      घर के अंदर पारिवारिक जीवन

Now I'd like to get your thoughts on a **healthy baby.**
अब मैं **स्वस्थ बच्चे** के बारे में आपके विचार जानना चाहूंगी।

- How do you know when a newborn baby is healthy? (in the first month after birth)
  जब एक नवजन्मा बच्चा स्वस्थ है तब यह आपको कैसे पता चलता है? (पैदा होने के बाद पहले माह में)
    - What do you look for or expect to see?
      आप किन चीजों को देखती हैं या देखने की उम्मीद करती हैं?
- What do you think are important things to do to ensure a healthy newborn baby
  एक स्वस्थ नवजन्मा बच्चा सुनिश्चित करने के लिए, आपके ख्याल से कौन सी चीजें जरूरी हैं?
    - Healthcare
      स्वास्थ्य देखभाल
    - Diet / nutrition
      आहार/पोषण
    - Family life within the household
      घर के अंदर पारिवारिक जीवन

And now I'd like to get your thoughts on a **healthy family**.
और अब मैं **स्वस्थ परिवार** के बारे में आपके विचार जानना चाहूंगी।

- What does a healthy family look like?
  एक स्वस्थ परिवार कैसा दिखाई देता है?
    - How many children? Why this many?
    - What sexes of children? Why these sexes?
    - How many years between children? Why this much spacing?
    - How much time between marriage and first pregnancy? Why this much time?

- What do you think are important things to do to ensure a healthy family?
  आपके ख्याल से एक स्वस्थ परिवार सुनिश्चित करने के लिए कौन सी चीजें जरूरी हैं?
    - Money / finances
      धन/वित्त
    - Healthcare
      स्वास्थ्य देखभाल
    - Diet / nutrition
      आहार/पोषण
    - Respect for other family members
      परिवार के दूसरे सदस्यों के लिए आदर
    - Family planning
      परिवार नियोजन

5. FAMILY PLANNINGCONTEXT
परिवार नियोजन परिप्रेक्ष्य

Tell me how youdecide and control when to have chidren - starting from marriage to the last child the couple you intend to have.
मुझे बताइये कि आप कैसे यह निर्णय लेती और नियंत्रण करती हैं कि बच्चे कब करने हैं – शादी से शुरुआत करते हुए आखिरी बच्चे तक जो आप पति–पत्नी पैदा करना चाहते हैं।

- Do / did you use any methods to control when to have children? Which methods?
  बच्चे कब करने हैं, इस पर नियंत्रण करने के लिए क्या आप कोई उपाय करती हैं/करती थीं? कौन सेउपाय?
- Why do you prefer this / these methods?
  आप इस उपाय/इन उपायों को पसंद करती हैं?
    - What do you like most about this method
      इस उपाय के बारे में आपको कौन सी बात सबसे ज्यादा पसंद है?
        - Ability to access the method
          इस उपाय तक पहुंचने की क्षमता
        - Ease of using the method
          इस उपाय को इस्तेमाल करने की सहूलियत
        - Side effects of the method
          इस उपाय के अनुषंगी प्रभाव
        - Alignment of the method to my household's beliefs
          मेरे परिवार की धारणाओं के साथ इस उपाय का मेल खाना
        - Alignment of the method to my religion
          मेरे धर्म के साथ इस उपाय का मेल खाना
    - What do you like least about this method
      आपको इस उपाय के बारे में कौन सी बात सबसे कम पसंद है
        - Ability to access the method
          इस उपाय तक पहुंचने की क्षमता
        - Ease of using the method

        इस उपाय को इस्तेमाल करने की सहूलियत

        - Side effects of the method
          इस उपाय के अनुषंगी प्रभाव
        - Alignment of the method to my household's beliefs
          मेरे परिवार की धारणाओं के साथ इस उपाय का मेल खाना
        - Alignment of the method to my religion
          मेरे धर्म के साथ इस उपाय का मेल खाना

- When did you use this / these methods?
  कब आपने यह उपाय/ये उपाय इस्तेमाल किए थे?
    - Before the first child? Which method?
      पहले बच्चे से पहले? कौन सा उपाय?
    - Between children? Which method?
      बच्चों के बीच में? कौन सा उपाय?
    - After the last child? Which method?
      आखिरी बच्चे के बाद? कौन सा उपाय?
- Who in your household has the greatest influence on when to have children?
  आपके परिवार में किसकी इस बात के बारे में सबसे ज्यादा चलती है कि बच्चे कब करने हैं?
- Who in your household has the greatest influence on how many children to have?
  आपके परिवार में किसकी इस बात के बारे में सबसे ज्यादा चलती है कि कितने बच्चे करने हैं?
- Who in your household has the greatest influence on what birth control method to use?
  आपके परिवार में किसकी इस बात के बारे में सबसे ज्यादा चलती है कि कौन सा गर्भनिरोधक उपाय करना है?
- Did the ASHA talk to you about family planning? When?
  क्याASHA ने आपसे परिवार नियोजन के बारे में बातचीत की थी? कब?
    - What was discussed?
      क्या बातचीत की गई थी?
        - Methods? Which ones?
          उपाय? कौन से?
        - Benefits of family planning?
          परिवार नियोजन के फायदे?
- Has anyone else talked to you about family planning? If so, who?
  क्या किसी और ने आपसे परिवार नियोजन के बारे में बातचीत की है? यदि हां, तो कौन?

6. BEHAVIOR STAGE
व्यवहार का चरण

PRIMER<br>प्रारंभक<br><br>You mentioned that you have used (method x) / have not used birth control. I would like to hear about why you and your family used method X.<br>आपने बताया कि आपने (फला–फलां उपाय) किया था/गर्भनिरोधक उपाय नहीं किया था। मैं आपसे जानना चाहूंगी कि क्यों आपने और आपके परिवार ने फलां–फलां गर्भनिरोधक उपाय किया था?<br><br>• Who was involved in the decision?<br>इस निर्णया में कौन–कौन शामिल था?<br>o Hospital staff<br>अस्पताल के कर्मचारी<br>o ASHA<br>o Family members<br>परिवार के सदस्य<br>• What was discussed?<br>किन–किन बातों पर चर्चा की गई थी?<br>• What were you thinking at that time?<br>उस समय आप क्या सोच रही थीं?<br>o Didyou personally want to use birth control at this time?<br>क्या इस बार आप निजी तौर पर गर्भनिरोधक उपाय करना चाहती थीं?<br>▪ If so, why?<br>यदि हां, तो क्यों?<br>▪ If any - which method did you personally prefer to use?	

SUPPORTING **BELIEFS**<br>समर्थन करने वाली**धारणाएं**<br><br>What were the reasons why you used (method x) / did not use birth control at this particular time?<br>वह कौन से कारण थे जिनकी वजह से आपने इस खास समय पर (फलां–फलां उपाय) किया था/गर्भनिरोधक उपाय नहीं किया था?<br><br>• Health benefits<br>स्वास्थ्य संबंधी फायदे<br>o Side effects / safety<br>अनुषंगी प्रभाव/सुरक्षा	*Start with first open question ONLY.*<br>*केवल पहले खुले प्रश्न के साथ शुरुआत करें।*<br><br>*Use probes only if respondent isn't responsive.*<br>*छानबीन केवल तभी इस्तेमाल करें जब प्रत्युत्तरदाता उत्तर नहीं दे रही है।*

○ Effectiveness of method<br>उपाय की कार्यसाधकता<br>○ Sensitivity and enjoyment<br>संवेदनशीलता और आनंद<br>• Practical benefits<br>व्यावहारिक फायदे<br>○ Household **financial situation** / concerns<br>परिवार के **आर्थिक हालात**/चिंताएं<br>○ **Space** constraints / concerns<br>**जगह** की तंगी/चिंताएं<br>○ **Chidcare** support concerns<br>**बाल देखभाल** सहायता की चिंताएं<br>○ **Availability** of supplies / method<br>सप्लाई/उपाय की **उपलब्धता**<br>○ Ease / effort required to **use** the method<br>उपाय **करने** के लिए जरूरी सहूलियत/मेहनत<br>• Thoughts about how other people may react<br>इस बारे में सोचविचार कि शायद दूसरे लोग कैसे प्रतिक्रिया करेंगे<br>○ Respect for other family members<br>परिवार के दूसरे सदस्यों के लिए आदर<br>○ Religious views<br>धार्मिक विचार<br>○ Views of my caste<br>मेरी जाति के लोगों के विचार	*If you don't receive a response after probing, then use beliefs cards*<br>*अगर आपको छानबीन के बाद उत्तर नहीं मिलता है तो फिर धारणाओं के कार्डों को इस्तेमाल करें।*

CONTRARY **BELIEFS**<br>विपरीत **धारणाएं**<br><br>What are reasons why you might have considered a different approach to family planning (using or not using birth control)? Which method?<br>वह कौन से कारण थे जिनकी वजह से शायद आपने परिवार नियोजन के एक अलग उपाय पर विचार किया होता (गर्भनिरोधक उपाय करना या नहीं करना)? कौन सा उपाय?<br><br>• Health concerns<br>स्वास्थ्य संबंधी फायदे<br>○ Side effects / safety<br>अनुषंगी प्रभाव/सुरक्षा<br>○ Effectiveness of method<br>उपाय की कार्यसाधकता	*Start with first open question ONLY.*<br>*केवल पहले खुले प्रश्न के साथ शुरुआत करें।*<br><br>*Use probes only if respondent isn't responsive.*<br>*छानबीन केवल तभी इस्तेमाल करें जब प्रत्युत्तरदाता उत्तर नहीं दे रही है।*<br><br>*If you don't receive a response after probing,*

○ Sensitivity and enjoyment<br>संवेदनशीलता और आनंद<br>• Practical concerns<br>व्यावहारिक फायदे<br>○ Household **financial situation** / concerns<br>परिवार के **आर्थिक हालात**/चिंताएं<br>○ **Space** constraints / concerns<br>**जगह** की तंगी/चिंताएं<br>○ **Chidcare** support concerns<br>**बाल देखभाल** सहायता की चिंताएं<br>○ **Availability** of supplies / method<br>सप्लाई/उपाय की **उपलब्धता**<br>○ Ease / effort required to **use** the method<br>उपाय **करने** के लिए जरूरी सहूलियत/मेहनत<br>• Thoughts about how other people may react<br>इस बारे में सोचविचार कि शायद दूसरे लोग कैसे प्रतिक्रिया करेंगे<br>○ Respect for other family members<br>परिवार के दूसरे सदस्यों के लिए आदर<br>○ Religious views<br>धार्मिक विचार<br>○ Views of my caste<br>मेरी जाति के लोगों के विचार<br>Out of all of these reasons, what were the 3 that most influencedusing (method X) or not using birth control?<br>इन सभी कारणों में से, वह कौन से 3 कारण थे जिन्होंने (फलां–फलां उपाय) करने या गर्भनिरोधक उपाय नहीं करने पर सबसे अधिक असर डाला था?<br><br>Betweenthese3reasons, which 1was the strongest and most influencedusing (method X) or not using birth control?<br>इन 3 कारणों में से, कौन सा 1 कारण सबसे अधिक दमदार था और जिसने (फलां–फलां उपाय) करने या गर्भनिरोधक उपाय नहीं करने पर सबसे अधिक असर डाला था?	*then use beliefs cards*<br>*अगर आपको छानबीन के बाद उत्तर नहीं मिलता है तो फिर धारणाओं के कार्डों को इस्तेमाल करें।*

**INFLUENCER**<br>**प्रभावशाली व्यक्ति**<br><br>You mentioned (x reason). I would like to hear about how you came to that conclusion in detail.<br>आपने (फलां–फलां कारण) बताया है। मैं आपसे विस्तार से जानना चाहूंगी कि आप उस निर्णय पर कैसे पहुंची थीं।	*Moderator to reference top belief and capture questions below for it, before moving to second and third belief.*<br>*मध्यस्थ सबसे प्रमुख धारणा का जिक्र करेगी और उसके नीचे*

Who or what influenced this reason the most<br>उस कारण को किसने या किस बात ने सबसे अधिक प्रभावित किया था?<br><br>• Conversations with / instructions from / observations of / previous experienceswith:<br>के साथ बातचीत/के निर्देश/के निरीक्षण/के साथ पिछले अनुभवः<br>o Family members<br>परिवार के सदस्य<br>o Community members<br>समुदाय के सदस्य<br>o Healthcare workers<br>स्वास्थ्य देखभाल कार्यकर्ता<br>o Healthcare signs or advertising<br>स्वास्थ्य देखभाल के चिह्न या इश्तिहार<br><br>**WHEN** did you come to this conclusion?<br>उस निष्कर्ष पर आप **कब** पहुंची थीं?<br><br>• Before marriage / before pregnancy / during pregnancy / after birth<br>शादी से पहले/गर्भावस्था से पहले/गर्भावस्था के दौरान/बच्चा पैदा करने के बाद	*मौजूद प्रश्नों का उत्तर पूछेगी, फिर दूसरी और तीसरी धारणा पर जाएगी।*<br><br>*Moderator to capture when*<br>*मध्यस्थ पूछेगी कि कब*

**MOTIVATION**<br>**प्रेरणा**<br><br>Why do you think they wanted you to know or believe this?<br>आपके ख्याल से वे लोग क्यों चाहते थे कि आपको यह पता हो या आप इस पर विश्वास करें?<br><br>• Why would telling / showing you that be important to them?<br>आपको यह बताना/दिखाना उनके लिए क्यों जरूरी होगा?<br>• How would they benefit from you believing them?<br>आपके द्वारा उन पर विश्वास करने से उन्हें कैसे फायदा होगा?<br>• How would these benefits make them feel?<br>इन फायदों से उन्हें कैसा महसूस होगा?	

**RESPECT**<br>**आदर**<br><br>How much respect did you have for their opinions at that time?<br>उस समय उनकी रायों के लिए आपके मन में कितना आदर था?<br><br>• High / medium / low<br>उच्च / मध्यम / कम	

**INFORMED**<br>**जानकार**<br><br>How well informed did you think they were at that time about family planning?<br>आपके ख्याल से उस समय उन्हें परिवार नियोजन के बारे में कितने अच्छे से जानकारी थी?<br><br>• High / medium / low<br>उच्च / मध्यम / कम	

**BEST INTERESTS**<br>**सर्वश्रेष्ठ हित**<br><br>Did you think at that time they had your best interests in mind?<br>क्या आपको लगता है कि उस समय उनके मन में आपके सर्वश्रेष्ठ हित मौजूद थे?<br><br>• High / medium / low<br>उच्च / मध्यम / कम	*Moderator to repeat influencer, motivation, respect, informed, and best interest questions for second and third beliefs.*<br>*मध्यस्थ दूसरी और तीसरी धारणाओं के लिए प्रभावशाली व्यक्ति, प्रेरणा, आदर, जानकार और सर्वश्रेष्ठ हित के प्रश्नों को दोहराएगी।*

**STAGE DEBRIEF**<br>**चरण के बारे में डिब्रीफ**<br><br>• When you made the decision to use (method x) / or not use birth control most recently, **how interested** were you to learn about using birth control?<br>हाल ही में, जब आपने (फलां–फलां उपाय) करने / या गर्भनिरोधक उपाय नहीं करने का अपना पिछला निर्णय लिया था, तब गर्भनिरोधक उपाय करने के बारे में जानने में **आपकीकितनी दिलचस्पी** थी?	

• High / medium / low<br>उच्च / मध्यम / कम<br><br>When you made the decision to use(method x) / or not use birth control most recently, **how much control** did you feel you had over decisions regardingusing birth control?<br>हाल ही में, जब आपने (फलां–फलां उपाय) करने / या गर्भनिरोधक उपाय नहीं करने का अपना पिछला निर्णय लिया था, तब गर्भनिरोधक उपाय करने के निर्णय पर आपको **अपना कितना नियंत्रण महसूस हुआ** था?<br><br>• High / medium / low<br>• उच्च / मध्यम / कम<br><br>When you made the decision to use (method x) / or not use birth control most recently, **who in your household had the most control**over using birth control?<br>हाल ही में, जब आपने (फलां–फलां उपाय) करने / या गर्भनिरोधक उपाय नहीं करने का अपना पिछला निर्णय लिया था, तब गर्भनिरोधक उपाय करने पर **आपके परिवार में किसका सबसे अधिकनियंत्रण** था?<br><br>• MIL / father / woman / other<br>सास / पिता / महिला / अन्य	

7. FUTURE BEHAVIORSTAGE
भविष्य के व्यवहार का चरण

**PRIMER**<br>प्रारंभक<br><br>**If your family were to decide and controlwhen to have children in the future, would you consider a different approach to family planning (using or not using birth control)?**<br>**यदि आपके परिवार को यह निर्णय लेना और नियंत्रण करना हो कि भविष्य में बच्चे कब करने हैं तो क्या आप परिवार नियोजन के लिए एक उपाय करने (गर्भनिरोधक उपाय करना या नहीं करना) के बारे में सोचविचार करेंगी?**<br><br>**Which birth control method would you consider, if any? Why?**<br>आप किस गर्भनिरोधक उपाय, यदि कोई, पर सोचविचार करेंगी? क्यों?<br><br>•	

**BELIEFS**<br>धारणाएं<br><br>What arethe main reasons why?<br>मुख्य कारण क्या थे, क्यों?<br><br>• Health benefits / concerns<br>स्वास्थ्य संबंधी फायदे<br>o Side effects / safety<br>अनुषंगी प्रभाव / सुरक्षा<br>o Effectiveness of method<br>उपाय की कार्यसाधकता<br>o Sensitivity and enjoyment<br>संवेदनशीलता और आनंद<br>• Practical benefits / concerns<br>व्यावहारिक फायदे<br>o Household **financial situation** / concerns<br>परिवार के **आर्थिक हालात** / चिंताएं<br>o **Space** constraints / concerns<br>**जगह** की तंगी / चिंताएं<br>o **Chidcare** support concerns<br>**बाल देखभाल** सहायता की चिंताएं<br>o **Availability** of supplies / method<br>सप्लाई / उपाय की **उपलब्धता**<br>o Ease / effort required to **use** the method<br>उपाय **करने** के लिए जरूरी सहूलियत / मेहनत<br>• Thoughts about how other people may react<br>इस बारे में सोचविचार कि शायद दूसरे लोग कैसे प्रतिक्रिया करेंगे<br>o Respect for other family members<br>परिवार के दूसरे सदस्यों के लिए आदर<br>o Religious views<br>धार्मिक विचार<br>o Views of my caste<br>मेरी जाति के विचार<br>Out of all of these reasons, which 3 will most influence using or not using birth control in the future?<br>इन सभी कारणों में से, वह कौन से 3 कारण होंगे जो भविष्य में गर्भनिरोधक उपाय करने या नहीं करने पर सबसे अधिक असर डालेंगे?<br><br>Between these 3 reasons, which 1is the strongest and will most influence using or not using birth control in the future?	*Start with first open question ONLY.*<br>*केवल पहले खुले प्रश्न के साथ शुरुआत करें।*<br><br>*Use probes only if respondent isn't responsive.*<br>*छानबीन केवल तभी इस्तेमाल करें जब प्रत्युत्तरदाता उत्तर नहीं दे रही है।*<br><br>*If you don't receive a response after probing, then use beliefs cards*<br>*अगर आपको छानबीन के बाद उत्तर नहीं मिलता है तो फिर धारणाओं के कार्डों को इस्तेमाल करें।*

इन 3 कारणों में से, कौन सा 1 कारण सबसे अधिक दमदार होगा और जो गर्भनिरोधक उपाय करने या नहीं करने पर सबसे अधिक असर डालेगा?	

**INFLUENCER**<br>**प्रभावशाली व्यक्ति**<br><br>You mentioned (x reason). I would like to hear about how you came to that conclusion in detail.<br>आपने (फलां–फलां कारण) बताया है। मैं आपसे विस्तार से जानना चाहूंगी कि आप उस निर्णय पर कैसे पहुंची थीं।<br><br>Who or what influenced this reason the most<br>उस कारण को किसने या किस बात ने सबसे अधिक प्रभावित किया था?<br><br>• Conversations with / instructions from / observations of / previous experiences with:<br>के साथ बातचीत / के निर्देश / के निरीक्षण / के साथ पिछले अनुभवः<br>o Family members<br>परिवार के सदस्य<br>o Community members<br>समुदाय के सदस्य<br>o Healthcare workers<br>स्वास्थ्य देखभाल कार्यकर्ता<br>o Healthcare signs or advertising<br>स्वास्थ्य देखभाल के चिह्न या इश्तिहार<br><br>**WHEN** did you come to this conclusion?<br>उस निष्कर्ष पर आप **कब** पहुंची थीं?<br><br>• Before marriage / before pregnancy / during pregnancy / after birth<br>शादी से पहले / गर्भावस्था से पहले / गर्भावस्था के दौरान / बच्चा पैदा करने के बाद	*Moderator to reference top belief and capture questions below for it, before moving to second and third belief.*<br>*मध्यस्थ सबसे प्रमुख धारणा का जिक्र करेगी और उसके नीचे मौजूद प्रश्नों का उत्तर पूछेगी, फिर दूसरी और तीसरी धारणा पर जाएगी।*<br><br>*Moderator to capture when*<br>*मध्यस्थ पूछेगी कि कब*

**MOTIVATION**<br>**प्रेरणा**<br><br>Why do you think they wanted you to know or believe this?<br>आपके ख्याल से वे लोग क्यों चाहते थे कि आपको यह पता हो या आप इस पर विश्वास करें?	

• Why would telling / showing you that be important to them?<br>आपको यह बताना/दिखाना उनके लिए क्यों जरूरी होगा?<br>• How would they benefit from you believing them?<br>आपके द्वारा उन पर विश्वास करने से उन्हें कैसे फायदा होगा?<br>• How would these benefits make them feel?<br>इन फायदों से उन्हें कैसा महसूस होगा?	

**RESPECT**<br>**आदर**<br><br>How much respect do you have for their opinions?<br>उस समय उनकी रायों के लिए आपके मन में कितना आदर था?<br><br>• High / medium / low<br>उच्च/मध्यम/कम	

**INFORMED**<br>**जानकार**<br><br>How well informed do you think they are about family planning?<br>आपके ख्याल से उस समय उन्हें परिवार नियोजन के बारे में कितने अच्छे से जानकारी थी?<br><br>• High / medium / low<br>उच्च/मध्यम/कम	

**BEST INTERESTS**<br>**सर्वश्रेष्ठ हित**<br><br>Did you think at that time they had your best interests in mind?<br>क्या आपको लगता है कि उस समय उनके मन में आपके सर्वश्रेष्ठ हित मौजूद थे?<br><br>• High / medium / low<br>उच्च/मध्यम/कम	*Moderator to repeat influencer, motivation, respect, informed, and best interest questions for second and third beliefs.*<br>*मध्यस्थ दूसरी और तीसरी धारणाओं के लिए प्रभावशाली व्यक्ति, प्रेरणा, आदर, जानकार और सर्वश्रेष्ठ हित के प्रश्नों को दोहराएगी।*

STAGE DEBRIEF<br>चरण के बारे में डिब्रीफ<br><br>In the future, if considering decisionsregarding family planning, **how interested** will you be to learn about birth control options and methods?<br>भविष्य में, यदि परिवार नियोजन के बारे में निर्णय पर सोचविचार करना पड़े तो गर्भनिरोधक उपायों और विकल्पों के बारे में जानने में **आपकीकितनी दिलचस्पी** होगी?<br><br>• High / medium / low<br>उच्च / मध्यम / कम<br><br>In the future if considering decisions regarding family planning, **how much control**will you feel you have over the use of birth control?<br>भविष्य में, यदि परिवार नियोजन के बारे में निर्णय पर सोचविचार करना पड़े तो गर्भनिरोधक उपाय करने के बारे में **आपको कितना नियंत्रण महसूस** होगा?<br><br>• High / medium / low<br>• उच्च / मध्यम / कम<br><br>In the future if considering decisions regarding family planning, **who in your household will have the most control** over the use of birth control?<br>भविष्य में, यदि परिवार नियोजन के बारे में निर्णय पर सोचविचार करना पड़े तो गर्भनिरोधक उपाय करने के बारे में **आपके परिवार में किसका सबसे ज्यादा नियंत्रण** होगा?<br><br>• MIL / father / woman / other<br>सास / पिता / महिला / अन्य	

8. DECISIONSTAGE
निर्णय का चरण

PRIMER<br>प्रारंभक<br><br>Now I'd like you to go back in time to when you most recently decidedhow to **controlwhen to have children**and tell me about when you first planned / talked about / thought abouthow to do this.<br>अब हम अतीत में उस समय पर वापस जाते हैं जब आपने पिछली बार यह निर्णय लिया कि **बच्चे कब करने हैं, इसे कैसे नियंत्रित किया** जाए, और मुझे बताइये कि आपने कब पहले योजना बनाई थी / बातचीत की थी / सोचविचार किया था कि यह कैसे करना है।	*Moderator to capture **WHEN***<br>*मध्यस्थ पूछेगी कि **कब***

• Did you have thoughts about whether birth control was necessary or not?<br>क्या आपने सोचविचार किया था कि क्या गर्भनिरोधक उपाय जरूरी थे या नहीं?<br>• If so, about **WHEN**did this happen?<br>यदि हां, तो लगभग **कब** हुआ था?<br>o Before marriage, before pregnancy, during pregnancy, after birth<br>शादी से पहले, गर्भावस्था से पहले, गर्भावस्था के दौरान, बच्चा पैदा करने के बाद<br>• Who was involved in the discussion, planning or your thoughts:<br>योजना बनाने या आपके सोचविचार में कौन शामिल था:<br>o ANM, AWW, Nurses, ASHA<br>o Family members<br>परिवार के सदस्य<br>o Community members<br>समुदाय के सदस्य<br>• What was discussed or thought about?<br>किन बातों पर चर्चा की गई या सोचविचार किया गया था?<br>• What family planning methods did you consider?<br>आपने किन गर्भनिरोधक उपायों पर सोचविचार किया था?	

**BELIEFS**<br>**धारणाएं**<br><br>At that time when you most recently considered controlling when to have children, what were the main reasons why you thought it was best to use (method x) / not use not birth control?<br>उस समय जब आपने पिछली बार यह नियंत्रित करने के बारे में सोचविचार किया था कि बच्चे कब करने हैं, तब वह कौन से मुख्य कारण थे जिनकी वजह से आपने सोचविचार किया था कि (फलां–फलां उपाय) करना / गर्भनिरोधक उपाय नहीं करना सर्वश्रेष्ठ है?<br><br>• Health benefits / concerns<br>स्वास्थ्य संबंधी फायदे<br>o Side effects / safety<br>अनुषंगी प्रभाव / सुरक्षा<br>o Effectiveness of method<br>उपाय की कार्यसाधकता<br>o Sensitivity and enjoyment	*Start with first open question ONLY.*<br>*केवल पहले खुले प्रश्न के साथ शुरुआत करें।*<br><br>*Use probes only if respondent isn't responsive.*<br>*छानबीन केवल तभी इस्तेमाल करें जब प्रत्युत्तरदाता उत्तर नहीं दे रही है।*<br><br>*If you don't receive a response after probing, then use beliefs cards*<br>*अगर आपको छानबीन के बाद उत्तर नहीं मिलता है तो फिर*

संवेदनशीलता और आनंद<br>• Practical benefits / concerns<br>व्यावहारिक फायदे/चिंताएं<br>◦ Household **financial situation** / concerns<br>परिवार के **आर्थिक हालात**/चिंताएं<br>◦ **Space** constraints / concerns<br>**जगह** की तंगी/चिंताएं<br>◦ **Chidcare** support concerns<br>**बाल देखभाल** सहायता की चिंताएं<br>◦ **Availability** of supplies / method<br>सप्लाई/उपाय की **उपलब्धता**<br>◦ Ease / effort required to **use** the method<br>उपाय **करने** के लिए जरूरी सहूलियत/मेहनत<br>• Thoughts about how other people may react<br>इस बारे में सोचविचार कि शायद दूसरे लोग कैसे प्रतिक्रिया करेंगे<br>◦ Respect for other family members<br>परिवार के दूसरे सदस्यों के लिए आदर<br>◦ Religious views<br>धार्मिक विचार<br>◦ Views of my caste<br>मेरी जाति के विचार<br><br>At that time when you most recently considered controlling when to have children, what were the 3 reasons that most influenced using (method x) / not using not birth control?<br>उस समय जब आपने पिछली बार यह नियंत्रित करने के बारे में सोचविचार किया था कि बच्चे कब करने हैं, इन सभी कारणों में से, वह कौन से 3 कारण थे जिन्होंने (फलां–फलां उपाय) करने या गर्भनिरोधक उपाय नहीं करने पर सबसे अधिक असर डाला था?<br><br>Between these 3 reasons, which 1was the strongest and most influenced using (method x) / not using not birth control?<br>इन 3 कारणों में से, कौन सा 1 कारण सबसे अधिक दमदार था और जिसने (फलां–फलां उपाय) करने या गर्भनिरोधक उपाय नहीं करने पर सबसे अधिक असर डाला था?	*धारणाओं के कार्डों को इस्तेमाल करें।*

**INFLUENCER**<br>**प्रभावशाली व्यक्ति**<br><br>You mentioned (x reason). I would like to hear about how you came to that conclusion in detail.	*Moderator to reference top belief and capture questions below for it, before moving to*

आपने (फलां–फलां कारण) बताया है। मैं आपसे विस्तार से जानना चाहूंगी कि आप उस निर्णय पर कैसे पहुंची थीं।<br><br>Who or what influenced this reason the most<br>उस कारण को किसने या किस बात ने सबसे अधिक प्रभावित किया था?<br><br>• Conversations with / instructions from / observations of / previous experiences with:<br>के साथ बातचीत / के निर्देश / के निरीक्षण / के साथ पिछले अनुभव:<br>o Family members<br>परिवार के सदस्य<br>o Community members<br>समुदाय के सदस्य<br>o Healthcare workers<br>स्वास्थ्य देखभाल कार्यकर्ता<br>o Healthcare signs or advertising<br>स्वास्थ्य देखभाल के चिह्न या इश्तिहार<br><br>**WHEN** did you come to this conclusion?<br>उस निष्कर्ष पर आप **कब** पहुंची थीं?<br><br>• Before marriage / before pregnancy / during pregnancy / after birth<br>शादी से पहले / गर्भावस्था से पहले / गर्भावस्था के दौरान / बच्चा पैदा करने के बाद	*second and third belief.*<br>*मध्यस्थ सबसे प्रमुख धारणा का जिक्र करेगी और उसके नीचे मौजूद प्रश्नों का उत्तर पूछेगी, फिर दूसरी और तीसरी धारणा पर जाएगी।*<br><br>*Moderator to capture when*<br>*मध्यस्थ पूछेगी कि कब*

**MOTIVATION**<br>**प्रेरणा**<br><br>Why do you think they wanted you to know or believe this?<br>आपके ख्याल से वे लोग क्यों चाहते थे कि आपको यह पता हो या आप इस पर विश्वास करें?<br><br>• Why would telling / showing you that be important to them?<br>आपको यह बताना / दिखाना उनके लिए क्यों जरूरी होगा?<br>• How would they benefit from you believing them?<br>आपके द्वारा उन पर विश्वास करने से उन्हें कैसे फायदा होगा?<br>• How would these benefits make them feel?<br>इन फायदों से उन्हें कैसा महसूस होगा?	

**RESPECT**<br>**आदर**<br><br>How much respect did you have for their opinions at that time?<br>उस समय उनकी रायों के लिए आपके मन में कितना आदर था?<br><br>• High / medium / low<br>उच्च / मध्यम / कम	

**INFORMED**<br>**जानकार**<br><br>How well informed did you think they were at that time about family planning?<br>आपके ख्याल से उस समय उन्हें परिवार नियोजन के बारे में कितने अच्छे से जानकारी थी?<br><br>• High / medium / low<br>उच्च / मध्यम / कम	

**BEST INTERESTS**<br>**सर्वश्रेष्ठ हित**<br><br>Did you think at that time they had your best interests in mind?<br>क्या आपको लगता है कि उस समय उनके मन में आपके सर्वश्रेष्ठ हित मौजूद थे?<br><br>• High / medium / low<br>उच्च / मध्यम / कम	*Moderator to repeat influencer, motivation, respect, informed, and best interest questions for second and third beliefs.*<br>*मध्यस्थ दूसरी और तीसरी धारणाओं के लिए प्रभावशाली व्यक्ति, प्रेरणा, आदर, जानकार और सर्वश्रेष्ठ हित के प्रश्नों को दोहराएगी।*

**STAGE DEBRIEF**<br>**चरण के बारे में डिब्रीफ**<br><br>When you most recentlyconsidered**controllingwhen to have children**, **how interested** were you to learn about birth control options and methods?<br>हाल ही में पिछली बार जब आपने पिछली बार **यह नियंत्रित करने के बारे में सोचविचार किया था कि बच्चे कब करने हैं,** तब गर्भनिरोधक उपायों और विकल्पों के बारे में जानने में **आपकीकितनी दिलचस्पी** थी?<br><br>• High / medium / low	

उच्च / मध्यम / कम<br><br>When you most recently considered **controllingwhen to have children,how much control** did you feel you had overbirth control (usage for the eligible couple) decisions?<br>हाल ही में पिछली बार जब आपने पिछली बार **यह नियंत्रित करने के बारे में सोचविचार किया था कि बच्चे कब करने हैं,** तब गर्भनिरोधक उपाय करने (पात्र पति–पत्नी के लिए इस्तेमाल) के निर्णयों पर आपको **अपना कितना नियंत्रण महसूस हुआ** था?<br><br>• High / medium / low<br>• उच्च / मध्यम / कम<br><br>When you most recently considered **controllingwhen to have children,who in your household had the most control**over birth control decisions?<br>हाल ही में पिछली बार जब आपने पिछली बार **यह नियंत्रित करने के बारे में सोचविचार किया था कि बच्चे कब करने हैं,** तब गर्भनिरोधक उपाय करने पर **आपके परिवार में किसका सबसे अधिकनियंत्रण** था?<br><br>• MIL / father / woman / other<br>सास / पिता / महिला / अन्य	

9. ORIGIN STAGE
आरंभिक चरण

**PRIMER**<br>प्रारंभक<br><br>Now I would like you to go way back in time and ask was there ever a time when you felt differently about whether to use or not use birth control?<br>अब मैं चाहूंगी कि आप अतीत में वापस जायें और अपने आपसे पूछें कि क्या कभी ऐसा कोई समय था जब आपको गर्भनिरोधक उपाय करने या नहीं करने के बारे में कुछ अलग महसूस हुआ था?<br><br>For example, when we were younger - we felt differently about all sorts of topics. Maybe you had different thoughts about whether birth control was necessary or not, what methods might be best if any, or what someone else told you when you were younger.<br>मिसाल के तौर पर, जब हम जवान थे – हमें सभी तरह की बातों के बारे में अलग महसूस होता था। हो सकता है आपके इस बारे में अलग विचार रहे हों कि गर्भनिरोध जरूरी था या नहीं, अगर कोई गर्भनिरोधक उपाय करना है तो कौन से उपाय सर्वश्रेष्ठ रहेंगे, या जब आप जवान थीं तब किसी दूसरे ने आपको कुछ बताया हो।	

If so:<br>यदि हां:<br>• Did you previously think it was better to use birth controlor not? Why?<br>क्या आप पहले सोचती थीं कि गर्भनिरोधक उपाय करना या नहीं करना बेहतर है? क्यों?<br>If yes – which birth control method did you think you would use? Why?<br>यदि हां – तो आपने कौन सा गर्भनिरोधक उपाय करने का सोचा था? क्यों?	

**BELIEFS**<br>**धारणाएं**<br><br>What were the main reasons why you had **previously felt** this way?<br>आपके द्वारा पहले इस तरीके से सोचे जाने के लिए, कौन सी मुख्य वजहें थीं?<br><br>• Health benefits / concerns<br>स्वास्थ्य संबंधी फायदे<br>o Side effects / safety<br>अनुषंगी प्रभाव / सुरक्षा<br>o Effectiveness of method<br>उपाय की कार्यसाधकता<br>o Sensitivity and enjoyment<br>संवेदनशीलता और आनंद<br>• Practical benefits / concerns<br>• व्यावहारिक फायदे / चिंताएं<br>o Household **financial situation** / concerns<br>परिवार के **आर्थिक हालात** / चिंताएं<br>o **Space** constraints / concerns<br>**जगह** की तंगी / चिंताएं<br>o **Chidcare** support concerns<br>**बाल देखभाल** सहायता की चिंताएं<br>o **Availability** of supplies / method<br>सप्लाई / उपाय की **उपलब्धता**<br>o Ease / effort required to **use** the method<br>उपाय **करने** के लिए जरूरी सहूलियत / मेहनत<br>• Thoughts about how other people may react<br>इस बारे में सोचविचार कि शायद दूसरे लोग कैसे प्रतिक्रिया करेंगे<br>o Respect for other family members	*Start with first open question ONLY.*<br>*केवल पहले खुले प्रश्न के साथ शुरुआत करें।*<br><br>*Use probes only if respondent isn't responsive.*<br>*छानबीन केवल तभी इस्तेमाल करें जब प्रत्युत्तरदाता उत्तर नहीं दे रही है।*<br><br>*If you don't receive a response after probing, then use beliefs cards*<br>*अगर आपको छानबीन के बाद उत्तर नहीं मिलता है तो फिर धारणाओं के कार्डों को इस्तेमाल करें।*

परिवार के दूसरे सदस्यों के लिए आदर<br>○ Religious views<br>धार्मिक विचार<br>○ Views of my caste<br>मेरी जाति के विचार<br><br>At that time, what were the 3 reasons that most influenced **why** you thought you would use (method x) / or would not use birth control<br>उस समय, वह कौन से 3 कारण थे जिन्होंने इस बात पर सबसे अधिक असर डाला था कि आप वह (फलां–फलां उपाय) करने या गर्भनिरोधक उपाय नहीं करने के बारे में **क्यों** सोच रही थीं?<br><br>Between these 3 reasons, which 1was the strongest and most influenced **why** you thought you would use (method x) / or would not use birth control?<br>इन 3 कारणों में से, कौन सा 1 कारण सबसे अधिक दमदार था और जिसने इस बात पर सबसे अधिक असर डाला था कि आप वह (फलां–फलां उपाय) करने या गर्भनिरोधक उपाय नहीं करने के बारे में **क्यों** सोच रही थीं?	

**INFLUENCER**<br>**प्रभावशाली व्यक्ति**<br><br>You mentioned (x reason). I would like to hear about how you came to that conclusion in detail.<br>आपने (फलां–फलां कारण) बताया है। मैं आपसे विस्तार से जानना चाहूंगी कि आप उस निर्णय पर कैसे पहुंची थीं।<br><br>Who or what influenced this reason the most<br>उस कारण को किसने या किस बात ने सबसे अधिक प्रभावित किया था?<br><br>• Conversations with / instructions from / observations of / previous experiences with:<br>के साथ बातचीत/के निर्देश/के निरीक्षण/के साथ पिछले अनुभव:<br>○ Family members<br>परिवार के सदस्य<br>○ Community members<br>समुदाय के सदस्य<br>○ Healthcare workers<br>स्वास्थ्य देखभाल कार्यकर्ता<br>○ Healthcare signs or advertising	*Moderator to reference top belief and capture questions below for it, before moving to second and third belief.*<br>*मध्यस्थ सबसे प्रमुख धारणा का जिक्र करेगी और उसके नीचे मौजूद प्रश्नों का उत्तर पूछेगी, फिर दूसरी और तीसरी धारणा पर जाएगी।*<br><br>*Moderator to capture when*<br>*मध्यस्थ पूछेगी कि कब*

स्वास्थ्य देखभाल के चिह्न या इश्तिहार<br><br>**WHEN** did you come to this conclusion?<br>उस निष्कर्ष पर आप **कब** पहुंची थीं?<br><br>• Before marriage / before pregnancy / during pregnancy / after birth<br>शादी से पहले/गर्भावस्था से पहले/गर्भावस्था के दौरान/बच्चा पैदा करने के बाद	

**STAGE DEBRIEF**<br>**चरण के बारे में डिब्रीफ**<br><br>When you had these different thoughts, **how interested** were you to learn about birth control options and methods?<br>जब आपके मन में ये अलग विचार थे, जब गर्भनिरोधक उपायों और विकल्पों के बारे में जानने के लिए आपकी **कितनी दिलचस्पी थी**?<br><br>• High / medium / low<br>उच्च/मध्यम/कम<br><br>When you had these different thoughts, **how much control** did you feel you had over a decision touse or not use birth control?<br>जब आपके मन में ये अलग विचार थे, जब गर्भनिरोधक उपाय करने या नहीं करने के निर्णय पर आपका **कितना नियंत्रण था**?<br><br>• High / medium / low<br>• उच्च/मध्यम/कम<br><br>When you had these different thoughts, **who in your household had the most control** over using or not using birth control?<br>जब आपके मन में ये अलग विचार थे, जब गर्भनिरोधक उपाय करने या नहीं करने के निर्णय पर आपके परिवार में **किसका सबसे अधिकनियंत्रण था**?<br><br>• MIL / father / woman / other<br>सास/पिता/महिला/अन्य	

10. AWARENESS STAGE

जागरुकता का चरण

**INFLUENCER**<br>प्रभावशाली व्यक्ति<br><br>Has anyone or anything (poster, advertisement, observations,etc) told you **there are advantages** to using birth control to delay when a newly married couple first gets pregnant and to space out the arrival of their children by at least 2 years?<br>क्या किसी व्यक्ति ने या किसी चीज ने (पोस्टर, इश्तिहार, निरीक्षण आदि) ने आपको बताया था किएक नवविवाहित दम्पत्ति द्वारा पहली बार गर्भ धारण करने में देरी करने और अपने बच्चों के आने में कम से कम 2 साल का अंतर रखने के लिए वहाँ गर्भनिरोधक उपाय इस्तेमाल के करने **कई फायदे हैं?**<br><br>• ANM, AWW, ASHA, other<br>• Family members<br>परिवार के सदस्य<br>• Community members<br>समुदाय के सदस्य<br>• Posters or radio<br>पोस्टर या रेडियो<br>• Observations or experiences<br>निरीक्षण या अनुभव	**If no, skip to Awareness Stage Debrief and Journey Debrief and finish interview.**<br>**यदि नहीं, तो जागरुकता चरण के डिब्रीफ और यात्रा डिब्रीफ को छोड़ दीजिये और बातचीत को खत्म कीजिये।**<br><br>**If yes,** *Moderator to capture* ***WHEN&INFLUENCER***<br>**यदि हां,** तो मध्यस्थ पूछेगी कि ***कब और कौन सा प्रभावशाली व्यक्ति***

**BELIEFS**<br>धारणाएं<br><br>Going back to that point in time, what were yourthoughts you had **when you first learnt**there are advantages to using birth control to delay when a newly married couple first gets pregnant and to space out the arrival of their children by at least 2 years?<br>यदि अतीत में उस समय पर वापस जायें तो जब आपको **पहली बार पता चला** कि एक नवविवाहित दम्पत्ति द्वारा पहली बार गर्भ धारण करने में देरी करने और अपने बच्चों के आने में कम से कम 2 साल का अंतर रखने के लिए वहाँ गर्भनिरोधक उपाय इस्तेमाल करने के कई फायदे हैं तब आपके मन में क्या विचार थे?<br><br>• Health benefits / concerns<br>स्वास्थ्य संबंधी फायदे<br>○ Side effects / safety<br>अनुषंगी प्रभाव / सुरक्षा<br>○ Effectiveness of method<br>उपाय की कार्यसाधकता	*Start with first open question ONLY.*<br>*केवल पहले खुले प्रश्न के साथ शुरुआत करें।*<br><br>*Use probes only if respondent isn't responsive.*<br>*छानबीन केवल तभी इस्तेमाल करें जब प्रत्युत्तरदाता उत्तर नहीं दे रही है।*<br><br>*If you don't receive a response after probing, then use beliefs cards*<br>*अगर आपको छानबीन के बाद उत्तर नहीं मिलता है तो फिर*

  - Sensitivity and enjoyment
    संवेदनशीलता और आनंद
- Practical benefits / concerns
  व्यावहारिक फायदे/चिंताएं
  - Household **financial situation** / concerns
    परिवार के **आर्थिक हालात**/चिंताएं
  - **Space** constraints / concerns
    जगह की तंगी/चिंताएं
  - **Chidcare** support concerns
    बाल देखभाल सहायता की चिंताएं
  - **Availability** of supplies / method
    सप्लाई/उपाय की **उपलब्धता**
  - Ease / effort required to **use** the method
    उपाय **करने** के लिए जरूरी सहूलियत/मेहनत
- Thoughts about how other people may react
  इस बारे में सोचविचार कि शायद दूसरे लोग कैसे प्रतिक्रिया करेंगे
  - Respect for other family members
    परिवार के दूसरे सदस्यों के लिए आदर
  - Religious views
    धार्मिक विचार
  - Views of my caste
    मेरी जाति के विचार

At that time, what were the 3 reasons that most influenced **you when you first learnt** there are advantages to using birth control to delay when a newly married couple first gets pregnant and to space out the arrival of their children by at least 2 years?
उस समय, जब आपको **पहली बार पता चला** कि एक नवविवाहित दम्पत्ति द्वारा पहली बार गर्भ धारण करने में देरी करने और अपने बच्चों के आने में कम से कम 2 साल का अंतर रखने के लिए वहाँ गर्भनिरोधक उपाय इस्तेमाल करने के कई फायदे हैं तब वह कौन से 3 कारण थे जिन्होंने आपको सबसे अधिक प्रभावित किया था?

Between these 3 reasons, which 1was the strongest and most influenced **you when you first learnt** there are advantages to using birth control to delay when a newly married couple first gets pregnant and to space out the arrival of their children by at least 2 years?
उस समय, जब आपको **पहली बार पता चला** कि एक नवविवाहित दम्पत्ति द्वारा पहली बार गर्भ धारण करने में देरी करने और अपने बच्चों के आने में कम से कम 2 साल का अंतर रखने के लिए वहाँ गर्भनिरोधक उपाय इस्तेमाल करने के कई फायदे हैं तब इन 3 कारणों में से कौन सा 1 कारण सबसे दमदार था और जिसने आपको सबसे अधिक

धारणाओं के कार्डों को इस्तेमाल करें।

प्रभावित किया था?	

**INFLUENCER**<br>**प्रभावशाली व्यक्ति**	*Influencer already captured in primer section above*<br>*उपर्युक्त प्रारंभक खंड में प्रभावशाली व्यक्ति के बारे में पहले ही पूछा जा चुका है।*

**MOTIVATION**<br>**प्रेरणा**<br><br>Why do you think (influencer) wanted you to know or believe this?<br>आपके ख्याल से (प्रभावशाली व्यक्ति) आपको यह क्यों बताना या इस पर विश्वास क्यों करवाना चाहती थी?<br><br>• Why would telling / showing you that be important to them?<br>आपको यह बताना/दिखाना उनके लिए क्यों इतना जरूरी होगा?<br>• How would they benefit from you believing them?<br>उन पर आपके विश्वास करने से उन्हें कैसे फायदा होगा?<br>• How would these benefits make them feel?<br>इन फायदों से उनको कैसा महसूस होगा?	

**RESPECT**<br>**आदर**<br><br>At that time, how much respect did you have for their opinions?<br>उस समय, उनकी राय के लिए आपके मन में कितना आदर था?<br><br>• High / medium / low<br>उच्च/मध्यम/कम	

**INFORMED**<br>**जानकार**<br><br>At that time, how well informed did you think they are about family planning?<br>उस समय, आपके ख्याल से वे लोग परिवार नियोजन के बारे में कितने अच्छे से जानते थे?<br><br>• High / medium / low<br>उच्च / मध्यम / कम	

**BEST INTERESTS**<br>**सर्वश्रेष्ठ हित**<br><br>Did you think at that time they had your best interests in mind?<br>क्या आपको लगता है कि उनके मन में आपके सर्वश्रेष्ठ हित मौजूद थे?<br><br>• High / medium / low<br>उच्च / मध्यम / कम	*Moderator to repeat influencer, motivation, respect, informed, and best interest questions for second and third beliefs.*<br>*मध्यस्थ सबसे प्रमुख धारणा का जिक्र करेगी और उसके नीचे मौजूद प्रश्नों का उत्तर पूछेगी, फिर दूसरी और तीसरी धारणा पर जाएगी।*

**STAGE DEBRIEF**<br>**चरण के लिए डिब्रीफ**<br>At that time, **when you first learnt** there are advantages to using birth control to delay when a newly married couple first gets pregnant and to space out the arrival of their children by at least 2 years, **how interested** were you to learn aboutbirth control options and methods?<br>उस समय, जब आपको **पहली बार पता चला** कि एक नवविवाहित दम्पत्ति द्वारा पहली बार गर्भ धारण करने में देरी करने और अपने बच्चों के आने में कम से कम 2 साल का अंतर रखने के लिए वहाँ गर्भनिरोधक उपाय इस्तेमाल करने के कई फायदे हैं तब गर्भनिरोधक उपायों एवं विकल्पों के बारे में जानने में **आपकी कितनी दिलचस्पी** थी?<br><br>• High / medium / low<br>उच्च / मध्यम / कम<br><br>At that time,**when you learnt that you should use birth control, how much control** did you feel you had over a decision to use or not use birth control?<br>उस समय, **जब आपकोपता चलाकिआपको गर्भनिरोधक उपाय करने चाहिए,** तब आपको	

गर्भनिरोधक उपाय करने या नहीं करने के निर्णय पर **अपना कितना नियंत्रण** महसूस हुआ था?<br><br>• High / medium / low<br>उच्च / मध्यम / कम<br><br>At that time, **when you learnt that you should use birth control, who in your household had the most control**over the decision to use or not use birth control?<br>उस समय, **जब आपकोपता चलाकिआपको गर्भनिरोधक उपाय करने चाहिए,** तब गर्भनिरोधक उपाय करने या नहीं करने के निर्णय पर **आपके परिवार में किसका सबसे अधिक नियंत्रण** था?<br><br>• MIL / father / woman / other<br>सास / पिता / महिला / अन्य	

11. UNDERSTANDING THE BENEFITS STAGE
फायदों को समझने का चरण

**INFLUENCER**<br>**प्रभावशाली व्यक्ति**<br><br>Has anyone or anything (poster, advertisement, observations, etc) told you **about the BENEFITS**of using birth control to delay when a newly married couple first gets pregnant and to space out the arrival of their children by at least 2 years?<br>क्या किसी व्यक्ति ने या किसी चीज ने (पोस्टर, इश्तिहार, निरीक्षण आदि) ने आपको बताया था कि एक नवविवाहित दम्पत्ति द्वारा पहली बार गर्भ धारण करने में देरी करने और अपने बच्चों के आने में कम से कम 2 साल का अंतर रखने के लिए वहाँ गर्भनिरोधक उपाय इस्तेमाल करने के **कई फायदे हैं?**<br><br>• If so, what are the benefits you remember?<br>यदि हां, तो आपको कौन से फायदे याद हैं?<br><br>• If so, about **when** did this happen?<br>यदि हां, तो यह लगभग **कब** हुआ था?<br>○ Before marriage / before pregnancy / during pregnancy / after birth<br>शादी से पहले / गर्भावस्था से पहले / गर्भावस्था के दौरान / बच्चा पैदा होने के बाद	**If no, skip to Awareness Stage Debrief and Journey Debrief and finish interview.**<br>**यदि नहीं, तो जागरुकता चरण के डिब्रीफ और यात्रा डिब्रीफ को छोड़ दीजिये और बातचीत को खत्म कीजिये।**<br><br>**If yes,** *Moderator to capture* ***WHEN & INFLUENCER***<br>**यदि हां,** तो मध्यस्थ पूछेगी कि ***कब और कौन सा प्रभावशाली व्यक्ति***

**BELIEFS**<br>धारणाएं<br><br>Going back to that point in time, what were some thoughts you had when first learning **about the BENEFITS** of using birth control to delay when a newly married couple first gets pregnant and to space out the arrival of their children by at least 2 years?<br>यदि उस समय पर वापस जायें जब आपको पहली बार पता चला था कि एक नवविवाहित दम्पत्ति द्वारा पहली बार गर्भ धारण करने में देरी करने और अपने बच्चों के आने में कम से कम 2 साल का अंतर रखने के लिए वहाँ गर्भनिरोधक उपाय इस्तेमाल करने के**कई फायदे हैं,** तब आपके मन में क्या विचार थे?<br><br>• Health benefits / concerns<br>स्वास्थ्य संबंधी फायदे<br>o Side effects / safety<br>अनुषंगी प्रभाव / सुरक्षा<br>o Effectiveness of method<br>उपाय की कार्यसाधकता<br>o Sensitivity and enjoyment<br>संवेदनशीलता और आनंद<br>• Practical benefits / concerns<br>व्यावहारिक फायदे / चिंताएं<br>o Household **financial situation** / concerns<br>परिवार के **आर्थिक हालात** / चिंताएं<br>o **Space** constraints / concerns<br>**जगह** की तंगी / चिंताएं<br>o **Chidcare** support concerns<br>**बाल देखभाल** सहायता की चिंताएं<br>o **Availability** of supplies / method<br>सप्लाई / उपाय की **उपलब्धता**<br>o Ease / effort required to **use** the method<br>उपाय **करने** के लिए जरूरी सहूलियत / मेहनत<br>• Thoughts about how other people may react<br>इस बारे में सोचविचार कि शायद दूसरे लोग कैसे प्रतिक्रिया करेंगे<br>o Respect for other family members<br>परिवार के दूसरे सदस्यों के लिए आदर<br>o Religious views<br>धार्मिक विचार<br>o Views of my caste<br>मेरी जाति के विचार	*Start with first open question ONLY.*<br>*केवल पहले खुले प्रश्न के साथ शुरुआत करें।*<br><br>*Use probes only if respondent isn't responsive.*<br>*छानबीन केवल तभी इस्तेमाल करें जब प्रत्युत्तरदाता उत्तर नहीं दे रही है।*<br><br>*If you don't receive a response after probing, then use beliefs cards*<br>*अगर आपको छानबीन के बाद उत्तर नहीं मिलता है तो फिर धारणाओं के कार्डों को इस्तेमाल करें।*

Out of all of these reasons, what were the 3 that most influenced **you when first learning about the BENEFITS** of using birth control to delay when a newly married couple first gets pregnant and to space out the arrival of their children by at least 2 years?<br>उस समय, जब आपको **पहली बार पता चला** कि एक नवविवाहित दम्पत्ति द्वारा पहली बार गर्भ धारण करने में देरी करने और अपने बच्चों के आने में कम से कम 2 साल का अंतर रखने के लिए वहाँ गर्भनिरोधक उपाय इस्तेमाल करने के कई फायदे हैं तब वह कौन से 3 कारण थे जिन्होंने आपको सबसे अधिक प्रभावित किया था?<br><br>Between these 3 reasons, which 1was the strongest and most influenced **you when first learningabout the BENEFITS** of using birth control?<br><br>उस समय, जब आपको **पहली बार गर्भनिरोधक उपाय इस्तेमाल करने के फायदों के बारे में पता चला था** तब इन 3 कारणों में से कौन सा 1 कारण सबसे दमदार था और जिसने आपको सबसे अधिक प्रभावित किया था?	

**INFLUENCER**<br>**प्रभावशाली व्यक्ति**	*Already captured*<br>*पहले पूछा जा चुका है*

**MOTIVATION**<br>**प्रेरणा**<br><br>Why do you think (influencer) wanted you to know or believe this?<br><br>आपके ख्याल से (प्रभावशाली व्यक्ति) आपको यह क्यों बताना या इस पर विश्वास क्यों करवाना चाहती थी?<br><br>• Why would telling / showing you that be important to them?<br>आपको यह बताना/दिखाना उनके लिए क्यों इतना जरूरी होगा?<br>• How would they benefit from you believing them?<br>उन पर आपके विश्वास करने से उन्हें कैसे फायदा होगा?<br>• How would these benefits make them feel?<br>• इन फायदों से उनको कैसा महसूस होगा?	

**RESPECT**<br>**आदर**<br><br>At that time, how much respect did you have for their opinions?<br><br>उस समय, उनकी राय के लिए आपके मन में कितना आदर था?<br><br>• High / medium / low<br>उच्च / मध्यम / कम	

**INFORMED**<br>**जानकार**<br><br>At that time, how well informed do you think they were about family planning?<br>उस समय, आपके ख्याल से उन्हें परिवार नियोजन के बारे में कितने अच्छे से जानकारी थी?<br><br>• High / medium / low<br>उच्च / मध्यम / कम	

**BEST INTERESTS**<br>**सर्वश्रेष्ठ हित**<br><br>Did you think at that time they had your best interests in mind?<br><br>क्या आपको लगता है कि उस समय उनके मन में आपके सर्वश्रेष्ठ हित मौजूद थे?<br><br>• High / medium / low<br>उच्च / मध्यम / कम	*Moderator to repeat influencer, motivation, respect, informed, and best interest questions for second and third beliefs.*

**STAGE DEBRIEF**<br>**चरण के लिए डिब्रीफ**<br><br>When you first learnt about the BENEFITS of birth control, **how interested** were you to learn about family planning?<br>जब आपको गर्भनिरोध के फायदों के बारे में पहली बार पता चला था तब परिवार नियोजन के बारे में जानने में आपकी **कितनी दिलचस्पी** थी?<br><br>• High / medium / low<br>उच्च / मध्यम / कम<br><br>When you first learnt about the BENEFITS of birth control,**how**	

**much control** did you feel you had for decisions concerning family planning?<br>जब आपको गर्भनिरोध के फायदों के बारे में पहली बार पता चला था तब परिवार नियोजन के बारे में निर्णयों पर आपको अपना **कितना नियंत्रण महसूस** हुआ था?<br><br>• High / medium / low<br>उच्च / मध्यम / कम<br><br>When you first learnt about the BENEFITS of birth control,**who in your household had the most control**over family planning decisions?<br>जब आपको गर्भनिरोध के फायदों के बारे में पहली बार पता चला था तब परिवार नियोजन के बारे में निर्णयों पर **आपके परिवार में किसका सबसे अधिक नियंत्रण था?**<br><br>• MIL / father / woman / other<br>सास / पिता / महिला / अन्य	

12.JOURNEY DEBRIEF
यात्रा के लिए डिब्रीफ

Finally, I'd like you to reflect back on our conversation and tell me:
आखिर में, मैं चाहूंगी कि आप हमारी बातचीत पर फिर से गौर करें और मुझे बतायें:

- What was the most stressful aspect of deciding to use a birth control method or not?
  गर्भनिरोधक उपाय करने या नहीं करने का निर्णय लेने का सबसे तनावपूर्ण पहलू क्या था?
- What was the most stressful aspect of actually using or not using birth control?
  वास्तव में गर्भनिरोधक उपाय करने या नहीं करने का निर्णय लेने का सबसे तनावपूर्ण पहलू क्या था?
- What is one thing that would have made your experience using the birth control method better?
  वह कौन सी एक बात है जिसने गर्भनिरोधक उपाय करने के आपके अनुभव को बेहतर बना दिया होता?
- What is the one key thing that would make you consider a differentbirth control decision (using or not using)?
  वह कौन सी मुख्य बात है जो आपसेएक अलग गर्भनिरोधक उपाय (करने या नहीं करने) का निर्णय लेते समय सोचविचार करवाएगी?
- What is the one key thing that would make you consider using a different birth control method?
  वह कौन सी मुख्य बात है जो आपसे एक अलग गर्भनिरोधक उपाय करने का निर्णय लेते समय सोचविचार करवाएगी?
- What is the one key thing that would make you consider waiting longer to have your first child after marriage?
  वह कौन सी मुख्य बात है जो शादी के बाद अपना पहला बच्चा करने से पहले अधिक समय इंतजार करने के लिए आपसे सोचविचार करवाएगी?

- What is the one key thing that would make you consider spacing out children differently?
  वह कौन सी मुख्य बात है जो अपने बच्चों के बीच अलग तरीके से अंतर के लिए आपसे सोचविचार करवाएगी?
- Did you share your any of your thoughts or concerns with any front-line healthcare workers (an ASHA, or a nurse for example).
  क्या आपने अपने किसी विचार या चिंता को पहली पंक्ति के अपने किसी स्वास्थ्य देखभाल कार्यकर्ता के साथ साझा किया था (मिसाल के तौर पर, एक ASHA)
  - If yes, how did they respond / react?
    यदि हां, तो उन्होंने क्या जवाब दिया/कैसे प्रतिक्रिया की?
