# Supplementary material for: Understanding drivers of family planning in rural northern India: An integrated mixed-methods approach
Source: PLoS One. 2021 Jan 13;16(1):e0243854. doi: 10.1371/journal.pone.0243854 (PMC7806122; doi:10.1371/journal.pone.0243854)
Supplement: S5 Appendix — (DOCX) [file pone.0243854.s005.docx]

**Decision game scenarios related to FP**

| Scenario 1 |
| --- |
| Janani, Rekha and Seema are being counselled by their ASHAs for contraceptive usage.   1. Janani's ASHA encourages her to use Mala D. She talks about how using the pill gives her more control of when she would get pregnant. 2. Rekha's ASHA encourages her to keep condoms. The ASHA says that anyway, husbands make these decisions, so condoms would be the right choice. 3. Seema's ASHA encourages her to get the IUCD because it’s long term and the safest.   Which of them will use the contraceptive being suggested by ASHA? |
| Scenario 2 |
| Maya, Vani and Shakti have had 3 children each. Their respective ASHAs advise them to adopt contraception to avoid further pregnancies. But the three women do not like the idea.   1. Maya feels that children is a gift from God, so she should not prevent pregnancy 2. Vani has heard stories about harmful side effects of contraception on woman's body 3. Shakti feels that it is not her decision to make whether to use contraception or not, her husband should decide.   Who among the three will not adopt contraception? |
| Scenario 3 |
| Usha, Jaya and Lata are women with three children each. They have been advised by their respective ASHAs to get sterilized.   1. Usha's ASHA told her that too many pregnancies is harmful for women's health 2. Jaya's ASHA told her that babies born to mothers with too many children are born weaker and with health problems 3. Lata's ASHA told her that bringing up children is a big cost, and hence it is better to have small family   Who among the three will accept ASHA's advice for sterilization? |
