# Supplementary material for: Understanding drivers of family planning in rural northern India: An integrated mixed-methods approach
Source: PLoS One. 2021 Jan 13;16(1):e0243854. doi: 10.1371/journal.pone.0243854 (PMC7806122; doi:10.1371/journal.pone.0243854)
Supplement: S6 Appendix — (DOCX) [file pone.0243854.s006.docx]

**Discussion guides for post decision game discussions related to FP**

| Scenario 1 |
| --- |
| What is your experience with family planning? Have you heard anything about the experience of other womens in your village? Mother can control pregnancy with Mala-D - What do you think about it? Do most women adopt Mala-D? Why? Why not?  Rekha - condom, so that the husband can decide- what do you think about it? Do most women like condoms? Why? Why not?  Seema - Cooper-T to prevent pregnancy for long time - what do you think about it? Do most women use Copper-T? Why? Why not?  Who told you about contraception? What do you think? What harm can happen?  Do you use any contraception for family planning? What do you prefer and why? If you don’t use anything, what do you think is most appropriate for your needs? Why? What do you think most women around the village use?  Which contraceptive method do you feel is harmful or problematic? Have your any stories about harmful affects of contraceptives? Tell us about it.  परिवार नियोजन को लेकर आपका क्या अनुभव है? क्या अपने गांव की अन्य महिलाओं के अनुभव के बारे में कुछ सुना है? जननी माला-डी से गर्भधारण में नियंत्रण कर सकती है? क्यों? क्यों नहीं?  रेखा - कंडोम, ताकि पति ये फैसला ले सके- इसके बारे में आप क्या सोचती हैं? क्या ज्यादातर महिलाऐं कंडोम पसंद करती हैं? क्यों? क्यों नहीं?  सीमा - कॉपर-टी, ताकि लम्बे समय तक गर्भधारण न हो - इसके बारे में आप क्या सोचती हैं? क्या ज्यादातर महिलाऐं कॉपर-टी इस्तेमाल करती हैं? क्यों? क्यों नहीं?  आपको गर्भनिरोधक के बारे में किसने बताया? आपको क्या फायदा लगता है? क्या नुकसान हो सकता है?  क्या आप परिवार नियोजन के लिए किसी गर्भनिरोधक का उपयोग करते हैं? आप क्या पसंद करते हैं और क्यों? यदि आप कुछ भी उपयोग नहीं करते हैं, तो आपको क्या लगता है कि आपकी आवश्यकताओं के लिए सबसे उपयुक्त क्या है? क्यों? आपको क्या लगता है कि गाँव की आसपास की अधिकांश महिलाएँ इसका उपयोग करती हैं?  आपको कौन सा गर्भनिरोधक तरीका हानिकारक या समस्याग्रस्त लगता है? क्या गर्भनिरोधक के हानिकारक प्रभावों के बारे में आपकी कोई कहानी है? हमें इस बारे में बताइये। |
| Scenario 2 |
| Maya, who considers children to be the gift of God - what do you think about it?  Vani - who fears bad effects in the body - what do you think about it?  Shakti - who thinks this is the husband's decision - what do you think about it?  How many children do you want? How much does your husband want? How much does your mother in law want? Who will make the final decision?  माया - जो बच्चों को भगवान की देन मानती है - इसके बारे में आपका क्या सोचना है?  वाणी - जिसे शरीर में बुरे असर का डर है - इसके बारे में आपका क्या सोचना है?  शक्ति - जिसे लगता है की ये तो पति का फैसला है - इसके बारे में आपका क्या सोचना है?  आप कितने बच्चे चाहती हो? आपके पति कितने चाहते हैं? आपकी सास कितने चाहती हैं? अंतिम निर्णय कौन लेगा? |
| Scenario 3 |
| What do you know about sterilization or what is your experience?  Usha - Get sterilized for your health - Do women find it right?  Jaya - For the health of their children - Do women get sterilization done for the health of children? Why? Why not?  Lata - To save on household expenses - Do most women perform sterilization to save expenses?  Do you know anyone who’s gone through the sterlization operation? What was their experience and did they have any problems?  Have you thought about sterilisation for yourself? Who have you spoken to about it? When do you think would be a good time to get sterilized?  What do you think is the main reason women get sterilised? Do you think about costs and expenses when you think of how many kids you want?  नसबंदी के बारे में आप क्या जानती हैं या आपका क्या अनुभव रहा है?  उषा - अपनी सेहत के लिए नसबंदी कराएं - क्या महिलाओं को यह ठीक लगता है?  जया - अपने बच्चो की सेहत के लिए - क्या महिलाएं बच्चो की सेहत के लिए नसबंदी करवाती हैं? क्यों? क्यों नहीं?  लता - घर के खर्चों को बचाने के लिए - क्या ज्यादातर महिलाएँ खर्च बचाने के लिए नसबंदी कराती हैं?  क्या आप किसी को जानते हैं जिसने नसबंदी ऑपरेशन करवाया हो? उनका अनुभव क्या था और क्या उन्हें कोई समस्या हुई?  क्या आपने अपने लिए नसबंदी के बारे में सोचा है? आपने इसके बारे में किससे बात की है? आपको कब लगता है कि नसबंदी करवाने का एक अच्छा समय होगा?  आपको क्या लगता है कि महिलाओं को नसबंदी कराने का मुख्य कारण क्या है? जब आप सोचते हैं कि आप कितने बच्चे चाहते हैं तब क्या आप लागत और खर्च के बारे में सोचते हैं? |
