# Supplementary material for: Understanding drivers of family planning in rural northern India: An integrated mixed-methods approach
Source: PLoS One. 2021 Jan 13;16(1):e0243854. doi: 10.1371/journal.pone.0243854 (PMC7806122; doi:10.1371/journal.pone.0243854)
Supplement: S7 Appendix — (DOCX) [file pone.0243854.s007.docx]

**Regional variability in method mix in UP in 2016**

*
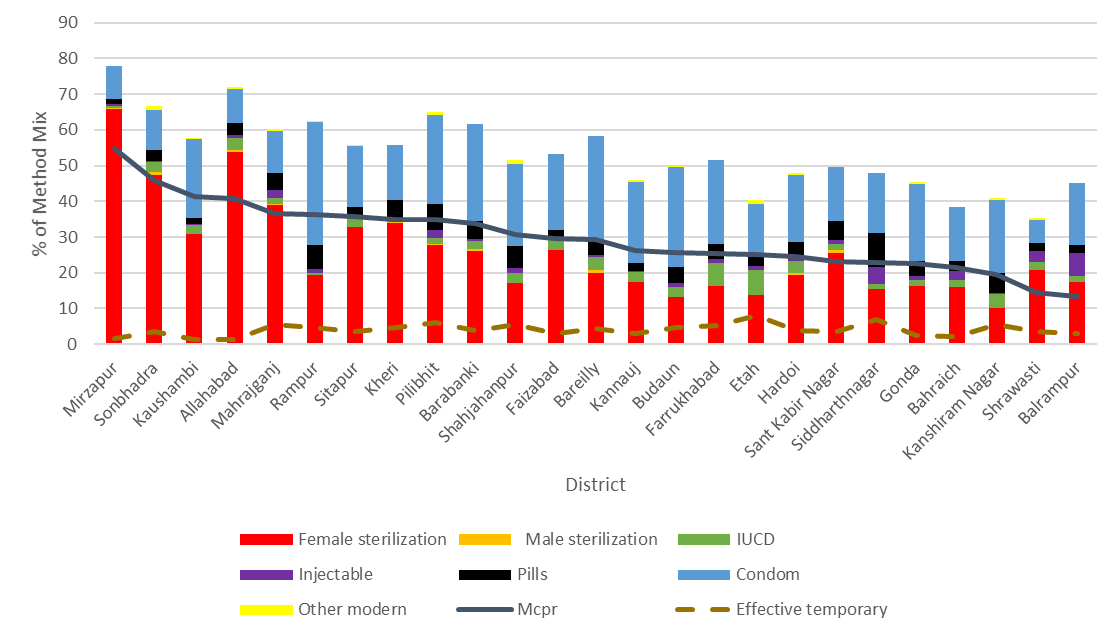
*

*The figure shows descriptive analysis of 2016 DLFPS (n= 13,182 married women ages 15-49). Despite observing little change in statewide methods mix over time, there is clear variability among districts in UP in both methods mix (bar segments) as well as the overall rate of modern contraceptive use (mCPR) (solid grey line). The use of effective temporary methods is low and more constant across districts. Districts are listed in order of decreasing mCPR. “Other modern” includes emergency contraception (ECP) and female condoms. “Effective Temporary” includes IUCD, injectable, ECP and pills (OCP).*
