# Supplementary figures and images for: Understanding drivers of family planning in rural northern India: An integrated mixed-methods approach
Source: PLoS One. 2021 Jan 13;16(1):e0243854. doi: 10.1371/journal.pone.0243854 (PMC7806122; doi:10.1371/journal.pone.0243854)

**The cascade for women who want to limit pregnancy versus those who want to space pregnancies**

**
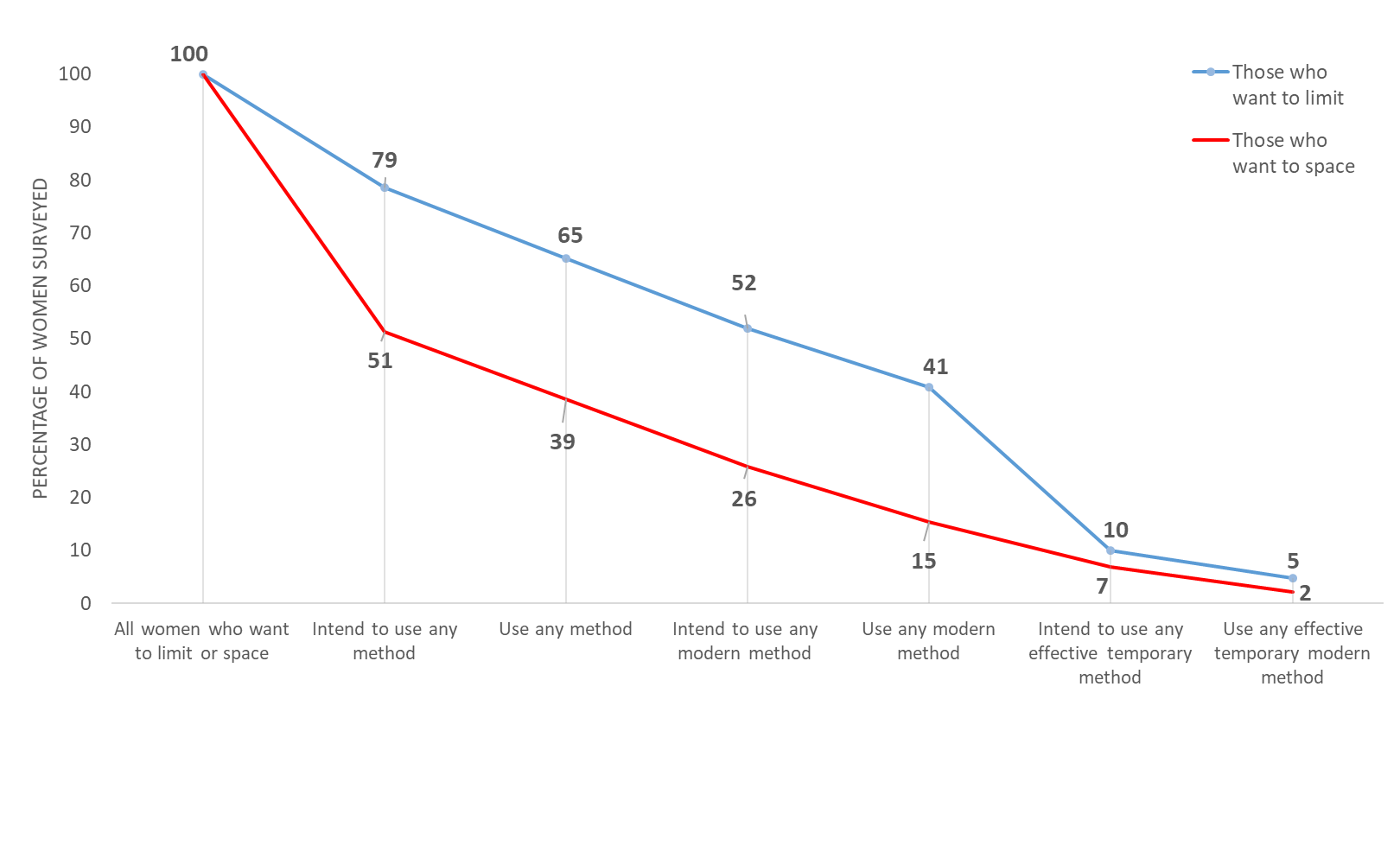
**

Supplement: S8 Appendix — (DOCX) [file pone.0243854.s008.docx]
