# Supplementary material for: Understanding drivers of family planning in rural northern India: An integrated mixed-methods approach
Source: PLoS One. 2021 Jan 13;16(1):e0243854. doi: 10.1371/journal.pone.0243854 (PMC7806122; doi:10.1371/journal.pone.0243854)
Supplement: S9 Appendix — (DOCX) [file pone.0243854.s009.docx]

**Contextual predictors of intention to use female sterilization, condoms, IUCDs, and pills**

|  | **Intent to Use Female Sterilization**  **OR (95% CI)** | **Intent to Use Condom**  **OR (95% CI)** | | **Intent to Use IUCD**  **OR (95% CI)** | | | **Intent to Use Pills**  **OR (95% CI)** |
| --- | --- | --- | --- | --- | --- | --- | --- |
| **Current** **Age** |  |  | |  | | |  |
| *Ref: <24* |  |  | |  | | |  |
| 25-29 | 2.13*** (1.57-2.88) | 0.96 (0.77-1.21) | | 0.90 (0.59-1.38) | | | 1.23 (0.87-1.76) |
| 30-34 | 3.08*** (2.27-4.19) | 0.75* (0.58-0.97) | | 1.01 (0.62-1.66) | | | 1.17 (0.8-1.71) |
| 35-49 | 2.75*** (2.03-3.72) | 0.37*** (0.28-0.49) | | 0.45* (0.26-0.76) | | | 0.55 (0.36-0.84) |
| **Age at Marriage** |  |  | |  | | |  |
| *Ref:<18* |  |  | |  | | |  |
| 18-20 | 0.85 (0.73-1.00) | 1.50*** (1.26-1.80) | | 1.30 (0.91-1.85) | | | 1.02 (0.77-1.35) |
| 21+ | 0.58** (0.39-0.85) | 1.66** (1.23 – 2.25) | | 1.22 (0.64-2.36) | | | 1.07 (0.61-1.87) |
| **Religion** |  |  | |  | | |  |
| *Ref: Hindu* |  |  | |  | | |  |
| Non-Hindu | 0.17*** (0.13-0.23) | 1.84*** (1.52-2.24) | | 0.69 (0.43-1.1) | | | 1.63** (1.19-2.23) |
| **Caste** |  |  | |  | | |  |
| *Ref: SC/ST* |  |  | |  | | |  |
| Non SC/ST | 0.81** (0.69-0.94) | 0.93 (0.77-1.12) | | 1.57* (1.05-2.36) | | | 1.11 (0.81-1.52) |
| **Literacy of Woman** |  |  | |  | | |  |
| *Ref: Illiterate* |  |  | |  | | |  |
| Literate | 0.92 (0.78-1.10) | 1.22* (1.03-1.46) | | 1.03 (0.69-1.53) | | | 1.19 (0.88-1.59) |
| **Literacy of Man** |  |  | |  | | |  |
| *Ref: Illiterate* |  |  | |  | | |  |
| Literate | 0.86 (0.74-1.01) | 1.34** (1.10-1.63) | | 1.19 (0.8-1.76) | | | 0.87 (0.65-1.17) |
| **SHG Member** |  |  | |  | | |  |
| *Ref: No* |  |  | |  | | |  |
| Yes | 1.27 (0.92-1.74) | 0.98 (0.70-1.38) | | 0.59 (0.25-1.37) | | | 0.72 (0.4-1.31) |
| **Wealth Quintile** |  |  | |  | | |  |
| *Ref: Poorest* |  |  | |  | | |  |
| Poor | 1.37** (1.11-1.69) | 0.96 (0.72-1.28) | | 1.04 (0.63-1.72 | | | 0.98 (0.65-1.48) |
| Middle | 1.62*** (1.31-2.00) | 0.81 (0.63-1.05) | | 0.54* (0.31-0.92) | | | 1.50 (0.99-2.29) |
| Rich | 1.59*** (1.27-2.01) | 1.18 (0.90-1.55) | | 0.67 (0.39-1.16) | | | 1.74* (1.12-2.72) |
| Richest | 1.74*** (1.34-2.24) | 1.42* (1.07-1.87) | | 0.75 (0.39-1.42) | | | 1.62* (1.02-2.6) |
| **Last had sex** |  |  | |  | | |  |
| *Ref: days ago* |  |  | |  | | |  |
| Weeks ago | 0.95 (0.77-1.16) | 0.95 (0.76-1.18) | | 1.40 (0.87-2.23) | | | 0.98 (0.69-1.41) |
| Months ago | 0.87 (0.73-1.03) | 0.73** (0.59-0.91) | | 1.28 (0.9-1.82) | | | 0.89 (0.66-1.19) |
| Years ago | 0.68 (0.43-1.08) | 0.32** (0.16-0.65) | | 1.67 (0.46-6.03) | | | 0.87 (0.39-1.94) |
| **Pregnant** |  |  | |  | | |  |
| *Ref: No* |  |  | |  | | |  |
| Yes | 6.685***(5.22-8.97) | 0.57*** (0.41-0.78) | | 2.01*** (1.21-3.34) | | | 2.77*** (1.92-3.99) |
| **Number of sons** |  |  | |  | | |  |
| *Ref: 0 sons* |  |  | |  | | |  |
| 1 son | 2.79*** (2.11-3.69) | 1.73*** (1.38-2.17) | | 1.42 (0.83-2.42) | | | 1.71** (1.16-2.51) |
| 2 sons | 5.09*** (3.84-6.75) | 1.57*** (1.23-2.00) | | 1.60 (0.86-2.96) | | | 2.65***(1.75-4) |
| 3 sons | 4.91*** (3.55-6.80) | 1.24 (0.90-1.70) | | 0.87 (0.39-1.94) | | | 2.53*** (1.51-4.22) |
| 4+ sons | 3.74*** (2.63-5.33) | 1.76** (1.21-2.55) | | 1.56 (0.67-3.66) | | | 3.03*** (1.75-5.24) |
| **Awareness of this method** |  |  | |  | | |  |
| *Ref: No*  Yes | 3.36** (1.55-7.30) | 3.26*** (1.86-5.71) | | 10.27*** (3.06-34.45) | | | 0.48 (0.21-1.1) |
| **Aware of any other modern**  **method** |  |  | |  | | |  |
| *Ref: No* |  |  | |  | | |  |
| Yes | 0.12*** (0.22-0.51) | 0.46* (0.23-0.90) | | 0.29 (0.03-3.04) | | |  |
| **Perceived Access of Method** |  |  | |  | | |  |
| *Ref: Easy* |  |  | |  | | |  |
| Hard | 0.36*** (0.30-0.44) | 1.29* (1.03-1.61) | | 0.22*** (0.14-0.34) | | | 0.79 (0.53-1.17) |
| Not aware | 0.12*** (0.10-0.14) | 0.60*** (0.49-0.74) | | 0.07*** (0.04-0.13) | | | 0.52*** (0.37-0.74) |
| **Perceived Access of Other mCPR** | |  |  | |  |  |  |
| *Ref: Easy* |  |  | |  | | |  |
| Hard | 1.57*** (1.30-1.91) | 0.90 (0.74-1.09) | | 2.43*** (1.54-3.84) | | | 1.17 (0.84-1.63) |
| Not aware | 2.33*** (1.89-2.86) | 0.96 (0.78-1.19) | | 2.09* (1.1-3.95) | | | 0.91 (0.6-1.38) |
| **Counselled by FLW on this method** |  |  | |  | | |  |
| *Ref: Yes*  No | 0.99 (0.64-1.53) | 1.65 *(1.00-2.71) | | 2.55** (1.43-4.55) | | | 3.51*** (2.1-5.85)) |
| **Counselled by FLW on other method** |  |  | |  | | |  |
| *Ref: Yes* |  |  | |  | | |  |
| No | 0.68 (0.42-1.08) | 0.72 (0.47-1.12) | | 0.85 (0.46-1.56) | | | 0.87 (0.58-1.29) |
| **Counselled by facility staff on this method** |  |  | |  | | |  |
| *Ref: No* |  |  | |  | | |  |
| At facility | 1.16 (0.79-1.70) | 1.18 (0.81-1.70) | | 2.23** (1.3-3.83) | | | 1.63* (1.02-2.61) |
| **Exposed to FP by media** |  |  | |  | | |  |
| *Ref: Yes* |  |  | |  | | |  |
| No | 0.99 (0.79-1.70) | 1.14 (0.97-1.34) | | 1.09 (0.75-1.58) | | | 1.01 (0.76-1.34) |
| **Informed about place of FP services** |  |  | |  | | |  |
| *Ref: Yes* |  |  | |  | | |  |
| No | 1.12(0.70-1.78) | 0.60 (0.36-1.01) | | 0.74 (0.4-1.35) | | | 0.73 (0.41-1.29) |
| **Received pills/condoms in the last year** |  |  | |  | | |  |
| *Ref: No* |  |  | |  | | |  |
| From ASHA/AWW | 0.82 (0.41-1.65) | 3.53*** (1.89-6.58) | | 0.91 (0.31-2.71) | | | 1.51 (0.73-3.12) |
| At Village Health and nutrition day | 0.58 (0.18-1.90) | 3.82*** (2.00-7.30) | | 0.71 (0.27-1.89) | | | 1.30 (0.52-3.24) |
| **Insulted/made to feel bad** |  |  | |  | | |  |
| *Ref: No* |  |  | |  | | |  |
| Yes | 1.13 (0.98-1.30) | 0.97 (0.83-1.15) | | 1.24 (0.89-1.73 | | | 1.05 (0.82-1.34) |
| _cons | 0.05*** (0.02-0.10) | 0.07*** (0.03-0.14) | | 0.01*** (0-0.05 | | | 0.03*** (0.01-0.09) |
| *P<0.05 **p<0.01 ***p<0.001 | | | | | | | |
