# Supplementary material for: Understanding drivers of family planning in rural northern India: An integrated mixed-methods approach
Source: PLoS One. 2021 Jan 13;16(1):e0243854. doi: 10.1371/journal.pone.0243854 (PMC7806122; doi:10.1371/journal.pone.0243854)
Supplement: S10 Appendix — (DOCX) [file pone.0243854.s010.docx]

**Percentage distribution of contextual factors among users and intenders by FP method**

| \|  \| **Female Sterilization** \| \| **Condom** \| \| **IUCD** \| \| **Pill** \| \| \| --- \| --- \| --- \| --- \| --- \| --- \| --- \| --- \| --- \| \| Users \| Intenders \| Users \| Intenders \| Users \| Intenders \| Users \| Intenders \| \| **Current** **Age** \| \| \| \| \| \| \| \| \| \| Ref: <24 \| 2.35 \| 25.31 \| 21.07 \| 30.45 \| 20.11 \| 42.39 \| 10.4 \| 26.78 \| \| 25-29 \| 13.94 \| 36.93 \| 32.03 \| 39.09 \| 27.72 \| 28.26 \| 32.21 \| 33.88 \| \| 30-34 \| 24.11 \| 26.56 \| 22.15 \| 16.05 \| 21.74 \| 22.83 \| 28.19 \| 17.49 \| \| 35-49 \| 59.6 \| 11.2 \| 24.75 \| 14.4 \| 30.43 \| 6.52 \| 29.19 \| 21.86 \| \|  \| *** \| \| *** \| \| *** \| \| *** \| \| \| **Age at Marriage** \| \| \| \| \| \| \| \| \| \| <18 \| 58.3 \| 68.98 \| 44.23 \| 39.09 \| 46.2 \| 46.74 \| 52.35 \| 64.48 \| \| 18-20 \| 24.77 \| 37.14 \| 44.3 \| 46.09 \| 40.22 \| 46.74 \| 37.58 \| 30.05 \| \| 21+ \| 3.63 \| 4.56 \| 11.47 \| 14.81 \| 13.59 \| 6.52 \| 10.07 \| 5.46 \| \|  \| *** \| \| ns \| \| ns \| \| ***** \| \| \| **Religion** \| \| \| \| \| \| \| \| \| \| Hindu \| 94.69 \| 94.19 \| 75.04 \| 71.6 \| 92.39 \| 77.17 \| 74.5 \| 68.85 \| \| Non-Hindu \| 5.31 \| 5.81 \| 24.96 \| 28.4 \| 7.61 \| 22.83 \| 25.5 \| 31.15 \| \|  \| ns \| \| ns \| \| *** \| \| ns \| \| \| **Caste** \| \| \| \| \| \| \| \| \| \| SC/ST \| 31.21 \| 38.38 \| 23.23 \| 25.51 \| 16.85 \| 23.91 \| 18.79 \| 26.23 \| \| Non SC/ST \| 68.79 \| 61.62 \| 76.77 \| 74.49 \| 83.15 \| 76.09 \| 81.21 \| 73.77 \| \|  \| ** \| \| ns \| \| ns \| \| ns \| \| \| **Literacy of Woman** \| \| \| \| \| \| \| \| \| \| Illiterate \| 69.61 \| 65.15 \| 46.54 \| 44.03 \| 40.76 \| 53.26 \| 56.38 \| 61.2 \| \| Literate \| 30.39 \| 34.85 \| 53.46 \| 55.97 \| 59.24 \| 46.74 \| 43.62 \| 38.8 \| \|  \| ns \| \| ns \| \| * \| \| ns \| \| \| **Literacy of Man** \| \| \| \| \| \| \| \| \| \| Illiterate \| 32.74 \| 36.31 \| 24.39 \| 23.87 \| 25.54 \| 21.74 \| 31.21 \| 40.44 \| \| Literate \| 67.26 \| 63.69 \| 75.61 \| 76.13 \| 74.46 \| 78.26 \| 68.79 \| 59.56 \| \|  \| ns \| \| ns \| \| ns \| \| * \| \| \| **SHG Member** \| \| \| \| \| \| \| \| \| \| No \| 93.51 \| 94.81 \| 95.45 \| 97.53 \| 96.74 \| 98.91 \| 96.64 \| 95.63 \| \| Yes \| 6.49 \| 5.19 \| 4.55 \| 2.47 \| 3.26 \| 1.09 \| 3.36 \| 4.37 \| \|  \| ns \| \| ns \| \| ns \| \| ns \| \| \| **Wealth Quintile** \| \| \| \| \| \| \| \| \| \| Poorest \| 17.06 \| 25.1 \| 12.19 \| 14.81 \| 15.22 \| 18.48 \| 9.4 \| 21.86 \| \| Poor \| 19.71 \| 24.69 \| 12.99 \| 13.99 \| 18.48 \| 23.91 \| 15.77 \| 16.94 \| \| Middle \| 21.4 \| 20.33 \| 16.45 \| 18.11 \| 15.22 \| 16.3 \| 16.11 \| 22.95 \| \| Rich \| 20.84 \| 17.63 \| 22.87 \| 20.16 \| 19.57 \| 22.83 \| 26.17 \| 19.67 \| \| Richest \| 20.99 \| 12.24 \| 35.5 \| 32.92 \| 31.52 \| 18.48 \| 32.55 \| 18.58 \| \|  \| *** \| \| Ns \| \| Ns \| \| *** \| \| \| **Last had sex** \| \| \| \| \| \| \| \| \| \| days ago \| 65.17 \| 56.64 \| 73.67 \| 47.33 \| 62.5 \| 47.83 \| 75.5 \| 40.98 \| \| weeks ago \| 13.84 \| 9.54 \| 14.94 \| 10.7 \| 16.85 \| 9.78 \| 11.74 \| 15.3 \| \| months ago \| 18.69 \| 32.78 \| 11.11 \| 37.86 \| 20.11 \| 40.22 \| 11.41 \| 40.44 \| \| years ago \| 2.3 \| 1.04 \| 0.29 \| 4.12 \| 0.54 \| 2.17 \| 1.34 \| 3.28 \| \|  \| *** \| \| *** \| \| ** \| \| ** \| \| \| **Number of sons** \| \| \| \| \| \| \| \| \| \| 0 son \| 2.5 \| 18.46 \| 16.02 \| 29.22 \| 13.59 \| 23.91 \| 9.06 \| 17.49 \| \| 1 son \| 19.36 \| 40.25 \| 37.81 \| 40.33 \| 36.41 \| 34.78 \| 30.54 \| 33.33 \| \| 2 sons \| 49.34 \| 25.52 \| 29.87 \| 18.93 \| 35.33 \| 31.52 \| 37.92 \| 28.96 \| \| 3 sons \| 19.2 \| 10.37 \| 10.03 \| 8.64 \| 8.15 \| 4.35 \| 14.43 \| 10.38 \| \| 4+ sons \| 9.6 \| 5.39 \| 6.28 \| 2.88 \| 6.52 \| 5.43 \| 8.05 \| 9.84 \| \|  \| *** \| \| *** \| \| ns \| \| ** \| \| \| **Perceived Access of Method** \| \| \| \| \| \| \| \| \| \| Easy \| 63.89 \| 16.8 \| 35.71 \| 13.99 \| 41.3 \| 21.74 \| 37.58 \| 12.57 \| \| Hard \| 27.63 \| 15.56 \| 35.71 \| 16.46 \| 35.87 \| 14.13 \| 39.6 \| 21.31 \| \| not aware \| 8.48 \| 67.63 \| 28.57 \| 69.55 \| 22.83 \| 64.13 \| 22.82 \| 66.12 \| \|  \| *** \| \| *** \| \| *** \| \| *** \| \| |
| --- | --- | --- | --- | --- | --- | --- | --- | --- | --- | --- | --- | --- | --- | --- | --- | --- | --- | --- | --- | --- | --- | --- | --- | --- | --- | --- | --- | --- | --- | --- | --- | --- | --- | --- | --- | --- | --- | --- | --- | --- | --- | --- | --- | --- | --- | --- | --- | --- | --- | --- | --- | --- | --- | --- | --- | --- | --- | --- | --- | --- | --- | --- | --- | --- | --- | --- | --- | --- | --- | --- | --- | --- | --- | --- | --- | --- | --- | --- | --- | --- | --- | --- | --- | --- | --- | --- | --- | --- | --- | --- | --- | --- | --- | --- | --- | --- | --- | --- | --- | --- | --- | --- | --- | --- | --- | --- | --- | --- | --- | --- | --- | --- | --- | --- | --- | --- | --- | --- | --- | --- | --- | --- | --- | --- | --- | --- | --- | --- | --- | --- | --- | --- | --- | --- | --- | --- | --- | --- | --- | --- | --- | --- | --- | --- | --- | --- | --- | --- | --- | --- | --- | --- | --- | --- | --- | --- | --- | --- | --- | --- | --- | --- | --- | --- | --- | --- | --- | --- | --- | --- | --- | --- | --- | --- | --- | --- | --- | --- | --- | --- | --- | --- | --- | --- | --- | --- | --- | --- | --- | --- | --- | --- | --- | --- | --- | --- | --- | --- | --- | --- | --- | --- | --- | --- | --- | --- | --- | --- | --- | --- | --- | --- | --- | --- | --- | --- | --- | --- | --- | --- | --- | --- | --- | --- | --- | --- | --- | --- | --- | --- | --- | --- | --- | --- | --- | --- | --- | --- | --- | --- | --- | --- | --- | --- | --- | --- | --- | --- | --- | --- | --- | --- | --- | --- | --- | --- | --- | --- | --- | --- | --- | --- | --- | --- | --- | --- | --- | --- | --- | --- | --- | --- | --- | --- | --- | --- | --- | --- | --- | --- | --- | --- | --- | --- | --- | --- | --- | --- | --- | --- | --- | --- | --- | --- | --- | --- | --- | --- | --- | --- | --- | --- | --- | --- | --- | --- | --- | --- | --- | --- | --- | --- | --- | --- | --- | --- | --- | --- | --- | --- | --- | --- | --- | --- | --- | --- | --- | --- | --- | --- | --- | --- | --- | --- | --- | --- | --- | --- | --- | --- | --- | --- | --- | --- | --- | --- | --- | --- | --- | --- | --- | --- | --- | --- | --- | --- | --- | --- | --- | --- | --- | --- | --- | --- | --- | --- | --- | --- | --- | --- | --- | --- | --- | --- | --- | --- | --- | --- | --- | --- | --- | --- | --- | --- | --- | --- | --- | --- | --- | --- | --- | --- | --- | --- | --- | --- | --- | --- | --- | --- | --- | --- | --- | --- | --- | --- | --- | --- | --- | --- | --- | --- | --- | --- | --- | --- | --- | --- | --- | --- | --- | --- | --- | --- | --- | --- | --- | --- | --- | --- | --- | --- | --- | --- | --- | --- | --- | --- | --- | --- | --- | --- | --- | --- | --- | --- | --- | --- | --- | --- | --- | --- | --- | --- | --- | --- | --- | --- | --- | --- | --- | --- | --- | --- | --- | --- | --- | --- | --- | --- | --- | --- | --- | --- | --- | --- | --- | --- | --- | --- | --- | --- | --- | --- | --- | --- | --- | --- | --- | --- | --- | --- | --- | --- | --- | --- | --- | --- | --- | --- | --- | --- | --- | --- | --- | --- | --- | --- | --- | --- | --- | --- | --- | --- | --- | --- | --- | --- | --- | --- | --- |

Chi-square test of association; *p<0.05 **p<0.01 ***p<0.001 ns p≥ 0.05
