# Supplementary material for: Understanding drivers of family planning in rural northern India: An integrated mixed-methods approach
Source: PLoS One. 2021 Jan 13;16(1):e0243854. doi: 10.1371/journal.pone.0243854 (PMC7806122; doi:10.1371/journal.pone.0243854)
Supplement: Attachment — Submitted filename: 20201026 Response to Reviewers_Plos FP paper.docx [file pone.0243854.s011.docx]

Jain et al.: "Understanding drivers of family planning in rural northern India: an integrated mixed-methods approach"

2020/10/26

**Response to reviewers**

Dear Dr Navaneetham,

Thank you very much for the review of our paper "Understanding drivers of family planning in rural northern India: an integrated mixed-methods approach" for your and the reviewers’ overall favorable assessment.

Your comments have greatly helped us sharpen the content of our manuscript, provide suitable level of details, and thus expand the paper’s potential usefulness to the field.

We have amended our manuscript as the reviewers have suggested. We have addressed all points raised by the editor and reviewers, and a detailed response to each point is given below. In addition to the specific lines we received comments on, we have made some additional but minor edits to the paper, in line with the reviewer comments, to bring further clarity to the key messages of the paper.

We have also included our updated cover letter with information on data availability and added additional supplementary materials for interview guides. The ethics statement has also been updated.

Again, we would like to thank the editors and reviewer for their comments, and we hope this manuscript will now be acceptable for publication.

Yours sincerely,

Sema K. Sgaier (Corresponding Author and on behalf of all authors), PhD

*Co-Founder & Executive Director,* Surgo Foundation

*Adjunct Assstant Professor*, Harvard T.H. Chan School of Public Health

*Affiliate Assistant Professor,* Global Health, University of Washington

**e:** [semasgaier@surgofoundation.org](mailto:semasgaier@surgofoundation.org)

Journal Requirements:

When submitting your revision, we need you to address these additional requirements.

1. Please ensure that your manuscript meets PLOS ONE's style requirements, including those for file naming. The PLOS ONE style templates can be found at

<https://journals.plos.org/plosone/s/file?id=wjVg/PLOSOne_formatting_sample_main_body.pdf> and

<https://journals.plos.org/plosone/s/file?id=ba62/PLOSOne_formatting_sample_title_authors_affiliations.pdf>

The paper has been formatted as per recommended guidelines.

2. Please include a copy of the interview guides used in the study, in both the original language and English, as Supporting Information, or include a citation if it has been published previously.

Interview guides have been added as supplementary files - S3, S4 and S6.

3. Please provide additional details regarding participant consent. In the ethics statement in the Methods and online submission information, please ensure that you have specified (1) whether consent was informed and (2) what type you obtained (for instance, written or verbal, and if verbal, how it was documented and witnessed). If your study included minors, state whether you obtained consent from parents or guardians. If the need for consent was waived by the ethics committee, please include this information.

Details around consent have now been added to the ethics statement and the beginning of the methods section of the paper as advised by the editorial team.

4. We note that you have indicated that data from this study are available upon request. PLOS only allows data to be available upon request if there are legal or ethical restrictions on sharing data publicly. For information on unacceptable data access restrictions, please see [(http://journals.plos.org/plosone/s/data-availability#loc-unacceptable-data-access-restrictions](http://journals.plos.org/plosone/s/data-availability#loc-unacceptable-data-access-restrictions).

In your revised cover letter, please address the following prompts:

a) If there are ethical or legal restrictions on sharing a de-identified data set, please explain them in detail (e.g., data contain potentially identifying or sensitive patient information) and who has imposed them (e.g., an ethics committee). Please also provide contact information for a data access committee, ethics committee, or other institutional body to which data requests may be sent.

We have added the details to justify choosing the option of making the data available upon request as per the directions of the ethical review board, Sigma IRB, New Delhi that approved the studies used in the paper. The following details have been added to the cover letter:

*De-identified datasets used in this paper can be made available upon request. The IRB board, Sigma IRB, New Delhi that approved all the studies directed the de-identified datasets should not publicly be made available as they contain sensitive and largely qualitative information on people’s private matters around sexual practices and family planning. However, the board has allowed interested individuals to send data requests to Dr U V Somayajulu, Member Secretary- Sigma IRB at Tel: (+ 91 11) 4619 5555 or email: somayajulu.uv@sigma-india.in.*

b) If there are no restrictions, please upload the minimal anonymized data set necessary to replicate your study findings as either Supporting Information files or to a stable, public repository and provide us with the relevant URLs, DOIs, or accession numbers. Please see <http://www.bmj.com/content/340/bmj.c181.long> for guidelines on how to de-identify and prepare clinical data for publication. For a list of acceptable repositories, please see <http://journals.plos.org/plosone/s/data-availability#loc-recommended-repositories>.

We will update your Data Availability statement on your behalf to reflect the information you provide.

[Note: HTML markup is below. Please do not edit.]

Reviewers' comments:

Reviewer's Responses to Questions

**Comments to the Author**

1. Is the manuscript technically sound, and do the data support the conclusions?

The manuscript must describe a technically sound piece of scientific research with data that supports the conclusions. Experiments must have been conducted rigorously, with appropriate controls, replication, and sample sizes. The conclusions must be drawn appropriately based on the data presented.

Reviewer #1: Partly

Reviewer #2: Partly

2. Has the statistical analysis been performed appropriately and rigorously?

Reviewer #1: I Don't Know

Reviewer #2: No

3. Have the authors made all data underlying the findings in their manuscript fully available?

The [PLOS Data policy](http://www.plosone.org/static/policies.action#sharing) requires authors to make all data underlying the findings described in their manuscript fully available without restriction, with rare exception (please refer to the Data Availability Statement in the manuscript PDF file). The data should be provided as part of the manuscript or its supporting information, or deposited to a public repository. For example, in addition to summary statistics, the data points behind means, medians and variance measures should be available. If there are restrictions on publicly sharing data—e.g. participant privacy or use of data from a third party—those must be specified.

Reviewer #1: Yes

Reviewer #2: Yes

4. Is the manuscript presented in an intelligible fashion and written in standard English?

PLOS ONE does not copyedit accepted manuscripts, so the language in submitted articles must be clear, correct, and unambiguous. Any typographical or grammatical errors should be corrected at revision, so please note any specific errors here.

Reviewer #1: Yes

Reviewer #2: Yes

5. Review Comments to the Author

Please use the space provided to explain your answers to the questions above. You may also include additional comments for the author, including concerns about dual publication, research ethics, or publication ethics. (Please upload your review as an attachment if it exceeds 20,000 characters)

Reviewer #1: Title- The title of the paper should mention the state of Uttar Pradesh (instead of saying northern India, since that’s the only state referred to in the paper.

Thank you for the suggestion. We believe that the approach, methods and implications described in this paper extend beyond Uttar Pradesh and are more broadly relevant for other geographies as well. Further, restricting the title to Uttar Pradesh might limit the audience this paper is able to reach. We have published other papers (e.g. [paper 1](https://www.ghspjournal.org/content/8/3/358.abstract), [paper 2](https://gh.bmj.com/content/5/8/e002437)) based on our research in Uttar Pradesh and have been able to use India more broadly in the title for the very reasons mentioned above. In light of these considerations, we would request that we are allowed to retain our original title.

Line 81- Total fertility rate for India is 2.2 (not 2.3).

Thank you, we have changed the total fertility rate to 2.2.

Para 81-93- This paragraph should bring in the fact that in 2017 the Indian Government introduced three new spacing methods, including injectable contraceptives, in the basket of contraceptive choices as a move to shift emphasis on spacing methods.

Thank you for your suggestion. We added this detail in the suggested paragraph.

Para 81-93- Given that this paper focuses on understanding the complex drivers of family planning uptake, the efforts made by the state of Uttar Pradesh, as described in the abstract, should also include demand generation efforts including any prominent social and behaviour change communication campaigns

Thank you for your suggestion. We have added the following details on the demand generation efforts to this paragraph.

*‘SIFPSA (State Innovations in Family Planning Services Project Agency), a joint venture of Government of India, USAID and Government of Uttar Pradesh has designed and implemented several mid- and mass-media campaigns to accelerate the government’s efforts for demand generation for family planning in the state [17]. Examples of these initiatives include a multi-media communication campaign called Aao Batein Karein with a primary focus on couples in the age group 17-25 currently not using contraception, Sarthi Sandeshwahini (a mobile video van for promotion of family planning in selected districts) and Sehat Sandesh Wahini (mobile video van project on RMNCH+A campaign). ‘*

Para 111-119- While condoms have been omitted from the categorization, they are important as they are the only spacing method involving men and their uptake is a critical indicator of male engagement in family planning.

Thank you for your suggestion. We agree that condoms, if used in the right manner, are an important and effective family planning method. However, from the data we collected in our qualitative research and our program learnings, we found the use of condoms to be quite inconsistent. Respondents mentioned that they did not use a condom all the times they had sex in a given cycle. This inconsistent pattern is associated with reduced effectiveness of the method as per WHO, which recommends a condom is correctly used every time one has sex. Further, in a lot of cases, women reported to not have control over ensuring its use, as highlighted in the paper. Inconsistent use of condoms makes it ineffective and are therefore removed from our categorisation of temporary effective methods, so that we can correctly present the usage levels of effective temporary methods in Uttar Pradesh. However, we have included your suggestion, and clarified and addressed this point in multiple places in the paper.

We have edited the paragraph (now 147 -155 in the revised manuscript) to say:

*‘Temporary effective modern contraception includes IUCDs, injectables, ECPs, and OCPs. Condoms,* ***an otherwise effective and crucial family planning method,*** *were omitted from the temporary effective category because our data showed that its use tends to be haphazard and inconsistent, and therefore not effective.’*

We have also edited paragraph 531 to 544 to say:

‘*Building intention will take time and cannot be achieved with quick interventions. There are, however, several ways to begin the process. Given the prevalence of fear of side-effects and infertility, risk perception around certain methods should be reduced and risk perception around unplanned pregnancies should be heightened. Women’s fear of side effects should be mitigated by addressing misconceptions (such as loss of fertility), providing strategies to mitigate real side effects, and helping women better understand their menstrual cycle. Messages should be targeted to fit the key barrier of specific methods. For example, fears of infertility caused by OCPs should be addressed differently than fears of bleeding caused by IUDs. Risk perception around unplanned pregnancies and other consequences of not practicing FP,* ***including inconsistent and haphazard use,*** *should be amplified, ideally by using narrative examples bringing risk into the familiar world and emphasizing modern contraceptives as the means of combatting said risk. Interventions should explicitly address contraceptive effectiveness of different methods to provide men and women with accurate and actionable information [38, 39]. Knowing which methods are more or less effective than others in preventing pregnancy can help individuals make more educated decisions on their contraceptive choices.’*

Further, we have given equal importance to condoms throughout the paper, by sufficiently analysing the method as part of all the different analyses presented in the paper. The need to actively involve men in FP has also been discussed in the paper.

**Results**

Para 257-263- It must be noted here that while the mCPR in UP hasn’t changed much between 2006-16, comparison of NFHS3 and NFHS4 does not show much change in India’s national mCPR between 2005-06 and 2015-16 either.

Thank you for pointing this out. We have added a line to revise the paragraph to say:

*‘Combined analysis of the NFHS and AHS reveals that the FP method mix in UP has changed little from 2006-2016 (Fig 1). Overall use of modern contraceptives (mCPR) in UP, however, has increased slowly since 2006 from 29.3% to 31.7% in 2016.* ***This trend of little change in mCPR is true for India overall as well, with mCPR decreasing slightly to 47.8% in 2015-16 from 48.5% in 2005-06 [16].*** *Despite consistency in method mix, the DLFPS data shows regional variability among high priority districts in UP in method use (S7 Appendix). The mCPR in Mirzapur is 55%, but it is only 13% in Balrampur. This finding suggests understanding the complex range of factors that influence uptake is very important’*

Para 327-338- What about female education/employment which would have a role in determining the decision making power of women and their ability to negotiate contraceptives. What is the reference for lines 333-335?

We included the variables female literacy and SHG membership in the multivariate regression models. SHG membership was not found to be significant in the models. Female literacy was only found to be a significant predictor of intention to use condoms, as shown in the table in S9 appendix.

The reference for lines 333-335 (and in fact the full section - *Influence of contextual factors varies by modern method)* is the multivariate regression models run on the DLFPS survey, details of which are mentioned in the Methods section. To further clarify the reference, we have made edits in the following lines in the paper:

Lines 203-206: ‘*In the FP cascade, to identify the predictors of intention to use of modern contraceptive methods, separate multivariate logistic regression models were* ***run on the DLFPS survey data*** *for the following dependent variables: had intention to use female sterilization, had intention to use pills, had intention to use condoms, and had intention to use IUCD.’*

Lines 375-377: *There are a number of other contextual factors that may drive intention to use a modern contraceptive method. Four multivariate logit regression models* ***were run on the DLFPS survey data*** *to determine contextual predictors of intention to use female sterilization, condoms, IUCDs, and pills.’*

Paragraph 394-415- The prevalence of myths and misconceptions around contraceptives as well as the limited uptake of contraceptives mentioned in previous paragraphs point towards the need to strengthen family planning counselling services which should also be highlighted**.**

Thank you for the suggestion. We have added this to the paragraph say:

*‘In addition to its expansive use of data, this study is, to our knowledge, the first to conduct a systematic assessment of FP behavioral drivers, both perceptual and contextual, and assess the influence of all stakeholders involved in the FP landscape (women, husbands, frontline workers, and mothers-in-law). These approaches allowed for a comprehensive exploration of FP drivers in Uttar Pradesh that provides insights to inform interventions. One key insight is the existence of a pronounced awareness-intention-action gap. The finding stresses the need to shift focus towards building intention, in addition to ensuring access. This research also reveals that in order to build intention to use modern contraceptives, interventions should also work towards changing perceptual drivers such as fear of infertility and side-effects, which is largely prevalent in the communities.* ***Current interventions, including family planning counselling services in communities and facilities need to be strengthened to address these gaps****. In addition, focus should be placed on engaging men and framing contraceptive use through economic arguments, as opposed to solely health-related justifications [35-37].’*

Overall- Though the paper touches upon the complex drivers of family planning in India, the background section needs to be strengthened further to present a stronger case for the family planning landscape in India where women do not have the ability to make their own fertility decisions as these decisions are made by the husband and family on her behalf. The second important point to be noted basis data from the fourth national family health survey is the high unmet need for family planning among women (which is defined as percentage of women in their reproductive age group who want to access contraception but are unable to do so due to various reasons). This is again an important indicator of women’s inability to take control of their fertility decisions. Greater emphasis on family planning counselling services and investments in behaviour change communication strategies are extremely important for demand generation.

The other point that needs to be brought out in the background section is UP’s large young population, who have distinct reproductive health needs which needs to be prioritized too.

Thank you for the detailed suggestions. We have substantially updated the introduction of the paper to incorporate both the reviewers’ comments on it.

**Reviewer #2:** Thank you for giving me the opportunity to review the article. The article is important and timely. Please find some suggestions. Hope those will be helpful and will strengthen the article for its contribution to future research on this topic.

We would like to extend our sincere thanks for providing such detailed and useful feedback. These have helped us improve our paper in several ways.

Specific comments:-

Abstract:

A disconnection between result and conclusion. The results showed that many factors acting at the individual and household level are responsible for low intention. All these factors are demand-side factors, but in conclusion, both demand-side and supply-side interventions have been highlighted, whereas the supply-side interventions and their implementations have not been studied or described, even using secondary sources.

Thank you for your comment. In the conclusion of the abstract, we stress upon the need for programs to also focus on demand side interventions that carefully address perceptual factors, over and above all the supply side interventions the programs are undertaking. In the introduction section of the paper in paragraph (94-124), we have provided several details of the supply side interventions being undertaken in Uttar Pradesh.

*‘As India’s largest state, Uttar Pradesh has undertaken many continued efforts to date to increase its modern contraceptive prevalence rate (mCPR). These efforts include increasing the number of IUCD and sterilization delivery points, building the capacity of healthcare providers through training in injectables and IUCDs, improved identification and support of couples with unmet need, and ensuring the availability of trained staff, commodities, drugs and equipment both in the community and the facility. The state has also created FP kits to distribute to newly-weds in rural areas, put up free condom boxes in strategic locations including hospitals, and raised financial incentives for sterilization procedures [17].’*

Introduction:

The introduction of the article is very generic. The authors argued for a holistic study for deeper understanding but did not explain how this will be helpful in the context of the family planning situation for Uttar Pradesh. Also provide some literature (may not necessarily be of family planning but other BCC interventions) how the holistic studies became helpful in drawing the programmatic recommendations and the outcome of the studies showed evidence of better programmatic outcome or impact.

Thank you for your comment. We have substantially updated the introduction to incorporate these and the 1st reviewers' comments.

The authors listed the programmatic efforts at the state level to increase the uptake of FP services. But how much those efforts have been successful? Please provide some figures on programmatic inputs. Everything in this paper is about the users. However, at the same time, it is important to know whether programmatic inputs are enough or not. Otherwise, it will seem like all programmatic efforts are complete; but the uptake is not happening only due to issues in the demand-side.

Thank you for the suggestion. We have further enhanced this section to more holistically mention all programmatic inputs, including both demand and supply side interventions that the state has pursued to increase modern contraceptive uptake. We mention that despite these set of efforts, the state falls short of its FP targets. Unfortunately, at the time of our research and analysis, this program had not carried out any scientific evaluation studies to be able to gauge the impact of these programmatic inputs directly on outcomes to be able to say if these efforts were successful or not. From the available information, what we were able to say is the state carried out a host of different demand and supply side interventions, which were not enough in terms of being able to meet the required levels of modern contraceptive uptake.

Method:

How authors decided the content of in-depth or immersion interviews. Were those based on the previous literature or from stakeholders’ interviews? What was the theoretical framework, and how that fits within the existing FP program in the state? Otherwise, it will be difficult to draw programmatic recommendations out of this study.

Thank you for asking this pertinent question. The in-depth immersion interviews were designed using findings from literature reviews and inputs from various stakeholder discussions. The inputs from the reviews and discussions were then structured using our conceptual framework, CUBES (described in the paper) to define the key areas of inquiry and design the interview guides.

We interacted with the state program managers for family planning within the Government of Uttar Pradesh and the Technical Support Unit (TSU) embedded in the Government of Uttar Pradesh tasked with supporting the state government to increase the efficiency, effectiveness and equity of the delivery of key RMNCH services including family planning. As part of our broader partnership with the TSU to support their RMMCH outcomes, we conducted rigorous exercises upfront to holistically map the logical framework of the program to identify the program goals, outcomes and outputs, and the gaps thereof.  We defined our research’s scope of work to explicitly address the program gaps to meet their goals. The stakeholder interviews were done to get programmatic insights and inputs on the performance of interventions along with the challenges and gaps the programs faced. As described in the introduction section of the paper, one big gap in the program was to understand what drives people’s behaviours around family planning uptake. The literature reviews and stakeholder discussions revealed several factors that could influence family planning uptake. These factors were structured using the CUBES framework to design the interview guides. The discussion guides included sections on intentions, actions, influencers and beliefs. The interview guides have been provided as supplementary files S3 Appendix.

Further, we have incorporated these details to modify paragraph 255 – 269 to say:

*‘****Literature reviews and stakeholder discussions were conducted to design the content of the in-depth immersion interviews, which revealed several factors that could influence family planning behaviors. These factors were structured using the CUBES framework to design the interview discussion guides [25]. The discussion guides (S3 Appendix) included sections on intentions, actions, influencers and beliefs.*** *In-depth immersion interviews of household members provided preliminary qualitative insights into the factors driving FP uptake in UP. In-depth Immersion interviews were approximately 90-minute-long, face to face, group, semi-structured interviews where households were asked a series of open-ended questions about their experiences and beliefs around FP [19]. Interviews were conducted in 9 blocks across 4 districts in UP. We interviewed those household members hypothesized to be primary influencers on FP. In addition to women and men, this included mothers-in-law (MIL) and sisters-in law (SIL). Women were interviewed separately from influencers (MIL, husband, and SIL) to allow them to respond independently. The interviews were analyzed using thematic analysis [32]. Themes included infrastructure, decision dynamics, social norms, past and current behaviors, beliefs, emotions, and stages of change. Findings from in-depth immersion interviews were then used to inform the design of the journey mapping and decision games.’*

Line 108: What does it mean by an immersion interview? How it is different from an in-depth interview? If methodologically not much difference between the two, suggesting using the term IDI because the use of that term is more popular.

Thank you for your comment. We have now changed the name to call it *In-depth Immersion Interviews*. We wish to retain the word immersion because we use a 3 methods integrated qualitative research. The in-depth immersion interviews were conducted first to uncover preliminary insights, which were used to design the more sophisticated methods of journey mapping and decision games. We have clarified this point in the paragraph to say:

*‘In order to probe deeper into the additional drivers and decision dynamics, the quantitative analysis was supplemented by de novo qualitative research on FP in UP using three methods: in-depth immersion interviews, journey mapping, and decision games.* ***These studies around FP were conducted as part of a larger study of RMNCH behaviors. As a first step, in-depth immersion interviews were conducted to provide preliminary insights into factors influencing RMNCH behaviours, which were consequently used to inform the design of journey mapping and decision games.*** *We conducted research on households, other household influencers, frontline workers, and facility staff involved in FP counseling and supply. For this paper, we analyzed the data from households and their influencers only. All qualitative data collection occurred in 2016. For this paper, we analyzed the data from households and their influencers only. All qualitative data collection occurred in 2016.’*

Line 115: Please clarify what does it mean by ‘safe method’. Does it mean ‘safe-period method’ or ‘standard day method’, if so kindly use either of the two terms because those are more standardized terms?

Thank you for your suggestion. We have replaced ‘safe method’ with standard days method.

Line 129: Remove the extra ‘period’ before (19).

Thank you pointing this out; removed.

Line 157: While providing the reference of a government document for high priority districts (reference #24) the authors wrote ‘Bhawan N’ as the author of the document. So far, my knowledge goes, it denotes “Nirman Bhawan”, which is a government administrative building in New Delhi. How can that be the author of a government document? Requesting authors to carefully check all references and avoid any such ambiguity and provide a proper citation for the said document. If any such document is available online, kindly provide the link for the readers in the reference as well.

Thank you for noting this. You are correct, this reference incorrectly cited a building as the other. We have corrected this reference accordingly and checked all other references to ensure that they are appropriately cited. We have also included the link in the reference.

Line 184: What does it mean by a 3-hour radius from state capital? I guess the authors mean a 3-hour driving distance. But that is a vague measurement. Suggesting providing an approximate distance or range in an absolute unit, kilometer.

Thank you for your suggestion. We have provided the range in kilometers.

Line 185: In place of ‘mixture’ suggesting ‘combination’.

Great suggestion. We have made the necessary edit.

Line 187-188. The authors selected the study block based on community literacy levels, the percentage of rural dwellers, and the percentage of community members coming from Scheduled Castes/Scheduled Tribes. The selection was based on whether the blocks had those indicators as high or low. Now the question comes what was considered as high and what as low. Please provide details in a table (maybe as an appendix) for those, otherwise it seems like the selection is highly purposive and not unbiased. For a qualitative study that is not a problem, but at least declares that it was a purposive selection if that was so. Also, which dataset provided the information for high and low percentages. I guess the authors took those from the census. If so, or anything else, please provide the reference.

Thank you for your suggestions. We have added the reference to the text and the details in the supplementary table, S1 Appendix.

Line 190-194: Kindly provide the reference of the data-set which have been used to calculate these indicators. Were these taken from NFHS or DLFPS?

Thank you for the suggestion. We used the block level indicators from HMIS and TSU’s program monitoring data. We have provided details of the data sets as well as methodology for determining what is ‘good’, ‘average’, or ‘poor (to address the next comment) in the S2 Appendix file.

Line 194-195: Please provide details of the methodology (maybe in the appendix) on how the authors determined what is ‘good’, ‘average’, or ‘poor’.

We have addressed this comment. The details, as mentioned above, have been provided in the S2 Appendix file.

Results:

Line 259 Please update the reference.

Thank you for your comment. The reference has been updated accordingly.

Line 267: In NFHS-4 both SDM and LAM have been considered as modern methods, not the traditional method. What adjustments did the authors take to make NFHS-4 data comparable with other survey rounds? Since DLFPS data only came from 25 HPDs authors should recalculate the indicators only among 25 HPDs of UP for other survey rounds to make those comparable with DLFPS estimations.

Thank you for the comments. AHS includes LAM as a traditional method; we have now subtracted the LAM numbers from the *Any Traditional Method* indicator from AHS to keep it consistent to NFHS’s definition. Further, we have removed the DLFPS numbers from the trend analysis based on your advice made under the 1st comment of Figures below.

DLFPS and NFHS-4 were done around the same time and therefore we agree it is better for us to remove the DLFPS numbers from this trend analysis, instead of restricting the other data sources to 25 HPDs only. Moreover, NFHS 3 doesn’t provide district level indicators. The qualitative studies were conducted in non HPD districts of UP as well, and therefore highlighting the situation at the macro state level will be more compelling for our full set of findings in terms of setting the context. The changes made are reflected in Figure 1

Line 286: Please update the reference.

Thank you for your comment. The reference has been updated accordingly.

Line 304: Please update the citation in the text. Requesting authors to carefully check the pdf file, generated for submission, before approving the submission.

Thank you for your comment. The in-text citation has been updated accordingly.

Line 320-322: I guess the authors changed the denominator in the second part of the sentence from the first part. Otherwise, it does not make a sense that “… 65% of women in the sample who wanted to limit, 79% intended to use any method, and 65% used any method…”. Please rephrase the whole sentence for both groups, who wanted to limit and to space.

Thank you for the suggestion. Yes 65% and 21% is the proportion of women in the sample who want to limit and space, respectively. We agree, including these numbers in the same sentence with changed denominators will be confusing for readers. We have now modified the text in the paragraph.

Line 327: From the previous paragraph (line 318-326) it seems authors want to pitch for bridging the gap between awareness and intention to use. The gap between awareness and intention is wider than the gap between intention and use. Therefore, suggesting exploring the contextual factor for intention to use rather than the use of methods, and that could be an original contribution from this analysis.

Thank you for the very useful suggestion. We have now run the models on those that had intent to use the specific methods and have updated the results accordingly. Further, to understand the drop off from intention to use, we did simple descriptive analyses to identify differences in various contextual factors between individuals that were currently using a method versus those had the intent to use a method but were not currently using it. These results have also been added to the paper.

Line 368: Please update the reference.

Thank you for your comment. The reference has been updated accordingly.

Line 419: Please update the reference.

Thank you for your comment. The reference has been updated accordingly.

Discussion:

Line 447 Are these factors responsible for low intention, or low use despite intentions?

Thank you for pointing this out. We have changed the language to say - *‘The qualitative data from interviews, journey mapping, and decision games provided more nuanced insights on the individual reasons for low intentions and uptake use not captured in the quantitative data.’* to keep it consistent with the language used at other places in the paper. Our analyses looked at gaps to intention and uptake as shown in figure 3. Sometimes similar factors came out as barriers along the different stages of change. However, from the quant data we know the biggest gap is in the awareness to intention stage of change, which is what we emphasize in the paper.

Line 449 If fear of infertility is high why those are not getting adopted by those who want to limit?

We found that individuals go for sterilisation once they have completed their desired number and gender of children and do not want more children. While making this decision, they are not concerned about the side effects from infertility. Fear of infertility is salient when considering temporary spacing methods. We have clarified this by specifying temporary methods while mentioning fear of infertility.

Line 449: Low-risk perception of consequences of FP non-use or low-risk perception of having additional child, or low perception of avoiding the additional child using FP. Please clarify.

Thank you for your suggestion. This finding is well described in the results section of the paper and now we have clarified this in the discussions section in lines 510-514 to say:

*‘First, it revealed that different beliefs and emotions played a large role in shaping uptake. Social norms against and shame to obtain modern contraceptives was widespread, while risk perception was high around side effects of methods – particularly fear of infertility from temporary methods,* ***but low around the consequences of not utilizing FP for having an unplanned additional or inadequately spaced child.’***

Line 450-453: In the Indian perspective there could be another pathway—those accept permanent method as their first FP method after reaching a certain number of children/sons. Requesting authors to consider this pathway as well.

Thank you for the great suggestion. We have added this information.

Line 462: How the fear of infertility and side-effects remained unaddressed in the current intervention? Were not those parts of the intervention when the current intervention was planned or were there any challenges while implementing those?

Thank you for the comment. These are perhaps being addressed to some extent in the counselling sessions that communities have with frontline workers and facility staff. However, we know that no specific training or guideline in terms of how to effectively address perceptual drivers is provided. We have now revised this to say:

*This research also reveals that in order to build intention to use modern contraceptives, interventions should also work towards changing perceptual drivers such as fear of infertility and side-effects, which is largely prevalent in the communities. Current interventions, including family planning counselling services in communities and facilities need to be strengthened to address these gaps.’*

Line 463: “Framing economic arguments for contraceptive use” could also raise some ethical issues as well. Raising a high number of children may put economic stress on the poor families but the economic argument may sound like it is ok for the ‘rich people’ to have as many children as they want because only they can afford it and poor families should have many children because they cannot afford!!! FP use is the right of the women and couples and the health program should make the methods accessible to those who need in a free, unbiased, and affordable way without any kind of coercion. Please reconsider the economic argument.

Thank you for your comments. In our study and literature (details below), we found that economics is a big part of the decision-making process in family planning. A lot of times, when individuals, especially men, decide to use family planning methods, it is because of considerations around the financial implications. The financial drivers are discussed in the results section of the paper. We are only suggesting that these economic considerations are also highlighted so that individuals can take informed decisions about their family planning goals. Our broader study on RMNCH in Uttar Pradesh also showed that men relate to financial arguments better than health related arguments.

Several studies in Africa (now referenced and cited in the paper) also bring out this point on economic consideration and suggest that financial benefits of financial planning should be discussed. Withers et al. say, “ Focusing on the advantages of family planning, namely financial benefits and reduced conflict among couples, could resonate with men.” Another study in Nigeria by Nte et al.  reported that the top benefit of family planning to the males as perceived by the women were reduction of financial burden. Also, Wambui et al. found that, “Likewise, the findings from the IDIs show that most of the couples nowadays discuss FP use because of economic hardship. People want to have manageable families they can afford to take care of.”

Line 479: Indian FP program does not include traditional methods so programmatically those methods do not get the endorsement. Why an intervention is required to discredit those methods? Kindly explain how discouraging the traditional methods are not going against the rights of the women or couples?

We completely accept your suggestions. We have removed the point around discrediting traditional methods. We have instead added a point on educating individuals on the relative effectiveness of different methods, which other studies have found to be a critical intervention. The following lines have been added.

*‘Interventions should explicitly address contraceptive effectiveness of different methods to provide men and women with accurate and actionable information [38, 39]. Knowing which methods are more or less effective than others in preventing pregnancy can help individuals make more educated decisions on their contraceptive choices.’*

Line 485: Emphasizing the financial benefits of FP for leveraging men can also result in coercion against women’s will for adopting FP methods like sterilization. Therefore, how sensible it is to talk about financial benefits to influence the husband’s decision for FP use.

Thank you for your comment. We have addressed this comment in our response to your comment on line 463 above. The financial element seems to be an important lever to engage men as other studies have also pointed out (which we have cited).

Line 488: Engaging the private sector is important for making the FP market more sustainable. I agree that the involvement of the private sector is important however, the authors' argument for private-sector engagement is not very convincing. Authors said because the drivers and barriers to contraceptive use are varied by person and method, FP offerings should adopt a private-sector approach. Not sure why a public sector approach will not be able to address those issues?

Also, how could a market-segmented approach simulate the uptake or stimulate the uptake? Please explain.

Thank you for your comments. In our recommendation here we do not recommend including the private sector versus or in addition to the public sector. We only suggest that the private sector approaches around acknowledging that individuals are different and their decisions are driven by different factors should be introduced in the current programs, so that individuals can be more precisely targeted. This is explained in the subsequent lines of 488. To further clarify, we have added a reference for the kind of private sector approaches we are referring to. Programs like (re)solve (funded by BMGF) in Bangladesh, Burkina Faso and Ethiopia are adopting similar market segmentation approaches in their family planning programs.

Line 497: What does it mean by ‘downward bias the coefficient’. Please explain.

We have changed the language to say  - depress the coefficient **towards 0** to clarify this. To explain this in any other meaningful way, it will become extremely technical and difficult for readers to follow. However, we have added a reference to a paper that describes this phenomenon.

Figures:

Please add a title to each figure.

A title is added to all figures.

Figure-1: Put X-axis on a time scale. Currently, it is categorial. Kindly justify why the results of the statewide surveys and the DLFPS are in the same graph. Did the estimates for NFHS and AHS have calculated only for those 25 HPDs? If not, requesting authors to do so, otherwise, remove the DLFPS estimates from the figure.

Thank you for your suggestion. We have removed the DLFPS data from this figure as explained in one of the previous replies. We have also formatted the X-axis to convert it to a time scale.

Figure-2: Requesting the authors to put the explanations of the cascade of 1, 2, and 3 as footnotes to the figure. I know the information is available in the text but a figure should be independent of the text—a stand-alone illustration.

We earlier had the footnotes for brackets - 1 and 2. We have now also added footnote 3, which was erroneously skipped. Thank you for pointing this out.

Figure-3: This figure and the whole analysis is heavily tilted towards the demand side. I was wondering why the supply-side issues and quality of care were not considered within this FP journey mapping. If the state has an issue of unavailability and access to contraceptive methods, it can create a barrier to translate the intention to use to actual use. Also, the poor quality of care can result in distrust of the health system and that can aggravate the fear of side-effects and fear of infertility which further acts as a barrier. Therefore,

Thank you for your comment. As mentioned in the methods section of the paper, the studies including the journey mapping have been holistically designed using the CUBES framework that in addition to the perceptual or internal factors also include structural or supply factors. Social and structural factors like *‘things and services from government are not of good quality*’ and *‘I do not have access to it’* were included in the study. However, the image of the journey map summarises the key enablers and barriers that emerged as drivers from our study. We did not find access and quality of care related issues to be a driving factor in people’s family planning decision journeys. This is why it is not reflected in the figure.

References:

Plos One uses the Vancouver style of referencing. Requesting the author to prepare the whole reference section following the style. Currently, there are several inconsistencies in the list of references. Some such inconsistencies are the following.

1. The journal names for reference #2, #3, and #18 were written differently. Even the same journal names have been differently formatted in different references e.g. #28 and #29.

Thank you for this note. We have reviewed our citations thoroughly to ensure consistency in formatting.

2. The references of AHSs all stat with parenthesis and the author names or organization names such as OotRGaCC or IIfPSIaM is very hard to figure out. Requesting authors to provide enough and standard information, required for reference so that readers and future researchers can benefit from this article.

Thank you for this suggestion. We have made edits to the citations as suggested.

3. Please format the reference for NFHS properly, a suggested citation for NFHS is provided in every NFHS report.

Thank you for this note. We had edited all NFHS citations to the suggested citation provided in the report.

6. PLOS authors have the option to publish the peer review history of their article ([what does this mean?](https://journals.plos.org/plosone/s/editorial-and-peer-review-process#loc-peer-review-history)). If published, this will include your full peer review and any attached files.

If you choose “no”, your identity will remain anonymous but your review may still be made public.

**Do you want your identity to be public for this peer review?** For information about this choice, including consent withdrawal, please see our [Privacy Policy](https://www.plos.org/privacy-policy).

Reviewer #1: **Yes:** Sanghamitra Singh

Reviewer #2: **Yes:** Arupendra Mozumdar

[NOTE: If reviewer comments were submitted as an attachment file, they will be attached to this email and accessible via the submission site. Please log into your account, locate the manuscript record, and check for the action link "View Attachments". If this link does not appear, there are no attachment files.]

While revising your submission, please upload your figure files to the Preflight Analysis and Conversion Engine (PACE) digital diagnostic tool, <https://pacev2.apexcovantage.com/>. PACE helps ensure that figures meet PLOS requirements. To use PACE, you must first register as a user. Registration is free. Then, login and navigate to the UPLOAD tab, where you will find detailed instructions on how to use the tool. If you encounter any issues or have any questions when using PACE, please email PLOS at figures@plos.org. Please note that Supporting Information files do not need this step.
