# Supplementary material for: Understanding drivers of family planning in rural northern India: An integrated mixed-methods approach
Source: PLoS One. 2021 Jan 13;16(1):e0243854. doi: 10.1371/journal.pone.0243854 (PMC7806122; doi:10.1371/journal.pone.0243854)
Supplement: Attachment — Submitted filename: 20201119 Response to Reviewers_Plos FP paper.docx [file pone.0243854.s012.docx]

Jain et al.: "Understanding drivers of family planning in rural northern India: an integrated mixed-methods approach"

2020/11/19

**Response to reviewers**

Dear Dr Navaneetham,

Thank you very much for the review of our paper "Understanding drivers of family planning in rural northern India: an integrated mixed-methods approach" for your and the reviewers’ overall favorable assessment.

We have further amended our manuscript based on the reviewers’ comments. We have addressed all points raised and a response to each point is given below. We have also submitted the other necessary documents.

Again, we would like to thank the editors and reviewer for their comments, and we hope this manuscript will now be acceptable for publication.

Yours sincerely,

Sema K. Sgaier (Corresponding Author and on behalf of all authors), PhD

*Co-Founder & Executive Director,* Surgo Foundation

*Adjunct Assstant Professor*, Harvard T.H. Chan School of Public Health

*Affiliate Assistant Professor,* Global Health, University of Washington

**e:** [semasgaier@surgofoundation.org](mailto:semasgaier@surgofoundation.org)

**6. Review** **Comments to the Author**

**Reviewer #1:** The article examines the important factors influencing demand generation in family planning. While the findings are relevant, some questions remain unanswered and some over-simplistic assumptions seem to have been made while analyzing the data. For example:

Lines 382-383: Talks about contraceptive use by religion. Have the authors tried to look at contraceptive uptake across religions AND wealth quintiles as well as literacy rates. This is a very important aspect that should be analyzed here.

Thank you for your comment. We fully agree that religion, wealth quintiles and literacy need to be all looked at, which is a part of the current analysis. The subsequent lines do discuss results around religions and wealth quintiles. You may reference the supplementary appendix S_9 for the full table for all variables included in the regression models. Demographic information of age, age at marriage, religion, caste, woman and husband’s literacy and wealth quintile have all been analyzed in the regression models.

Line 456 onwards: Were questions around risk perception on contraceptive side effects accompanied by questions around access to quality family planning counselling services? Again this is very important to understand. In fact the discussion section should also talk about provider bias which dissuades users from accessing contraceptive services, specially young people.

Great point. The research did investigate about access to family planning counselling. In the regression models, we found that counselling on specific methods by FLWs and facility staff was associated with higher intention to use pills, condoms and IUDs. While provider bias is an important point – something we’ve looked into as part of other research, it did not emerge as a factor in the research we did with households.

Overall: There should be a perspective on Uttar Pradesh's large young population, particularly the large adolescent population. In fact the state has the largest adolescent population in India. Their contraceptive needs are distinct, which also need to be catered to and the manuscript should include a mention of that.

Thank you for your comment. We had added the importance of catering to the youth given they form a large part of the total population in the introduction section of the paper. The results throughout discuss key FP patterns observed in younger men and women. However, while adolescents are a crucial and distinct segment in the population, we feel that writing about adolescent population in our paper would be we beyond the scope of this paper. Our research was done for married adult couples and the program was also focused on this sub population.

**Reviewer #2:** Thank you for giving me the opportunity to review the revised version of the article titled “Understanding drivers of family planning in rural northern India: an integrated mixed-methods approach” (PONE-D-20-21461R1). I also thank the authors for considering the comments in revising the manuscript. Please find some minor comments on this version of the manuscript. Hope those will be helpful.

Thank you for your excellent comments. The last round of comments incredibly helped us sharpen the paper. We have addressed all your following comments as well.

Specific comments: -


Introduction:

Line 114 The governments in India have taken a target-free approach for implementing the FP program. Therefore, suggesting replacing the word ‘target’ from this sentence with another suitable word.

Absolutely. We have now edited the line to remove the word ‘target’


Method:

Line 194 Requesting to add a short note on ASHA areas, maybe as a footnote, especially for the readers from outside India.

We have added a line to describe an ASHA area in the subsequent line since the journal doesn’t permit adding footnotes.

Line 210 Suggesting replacing ‘simple chi-square test’ with ‘chi-square test of independence’.

We have made the change. Thank you!


Results:

Line 345 Kindly include male sterilization among the list of modern contraceptives.

Thank you for catching the omission. We have added it.

Line 386 Suggesting rephrasing “Awareness of other methods was a negative predictor of intention to use female sterilization, and condoms.” I am not questioning the association, but awareness of other methods is probably not predicting the non-use of sterilization and condoms, it is something else. It is more possible that users of those two methods do not know any other methods. It raises the question of the quality of care received by those women; they were not told about other methods by the providers at the time of method adoption at least in the case of sterilization.

Great suggestion. We have now rephrased the sentence to say – ‘*Those that were not aware of other methods beyond female sterilization and condoms were more likely to have an intention to use female sterilization and condoms, respectively. This suggests that some women’s choice of these two methods is largely driven by the lack of awareness of other available methods.’*

Line 389 I don’t understand what does it mean by “perceived access to be hard for sterilization”. Suggesting the rephrase in a simpler language.

Sure. We have rephrased the language.

Line 396 Suggesting using “demographic characteristics” instead of “demographic mix”.

We have replaced the word as suggested.

Line 418 If women don’t have any power over the decision of condom use, it is a problem of women’s rights. What is the logic behind reporting the decision making of condom use as ‘hierarchical’? I am sorry if I misunderstood, but the use of the word ‘hierarchical’ in some way sounds like a justification for lack of decision-making power, as if a woman’s status is lower to her husband or other family members, and it’s like everyone (at least the people of the scientific community) all accept it that way. Do authors themselves perceive husbands are in a higher social status than women? If not, I suggest just report who is making the decision for condom use. If it is the husband or in-laws or any other family members, just report that without any justification, such that, it does not appear as if it is an obvious thing to happen. It is not acceptable in any condition that women are not in a position to make her own decision for using a contraceptive.

This is a very important comment. We agree the word hierarchical was not intended to imply any of the above. As suggested, we have simply mentioned that men are key decision makers for condoms.

Line 477 I would like to say this sentence about the usefulness of financial risk perception as a target for intervention should go to the discussion section of the paper leaving the result section only for findings and not to draw any programmatic recommendation in the result section.

Thank you! We have deleted this sentence.


Discussion:

Line 551 I agree that financial issue plays a big role in decision making of the couples especially the husbands on their fertility preference. But as I pointed out in my original review bringing the risk of financial issues in interventions could also raise some ethical issues. Putting the financial argument in program intervention for contraceptive use will disproportionately discourage the families of low socioeconomic strata to achieve their fertility goal. Therefore, I suggest that the authors should draw programmatic recommendations by addressing the consequences of financial risk on family health instead of including the issues of financial risk directly.

Thank you for reiterating the point. We agree with your suggestions and the alternate phrasing. We have rephrased to say – *‘For example, highlighting the consequences of financial risk on family health and wellbeing emphasizing the financial benefits of FP could be a key message to leverage for men.’*

Line 564 It depends on which model we are talking about. If we are dealing with logistic models shifting the coefficient towards 0 is not a bad thing. Suggesting changing “…the coefficients towards 0” into “…the coefficients towards non-significance”.

The literature doesn’t suggest shifting coefficients towards non-significance. It basically is unable to capture the true effect sizes in case of omitted variables and in logistic regression essentially reduces them towards 0. We have decided to exclude this sentence as this implication is too technical. We think the limitation that perceptual factors were not measured and therefore not included in the quantitative models should suffice.

Figures:

Please add a title to each figure.

Per the PLOS One guidelines, we have bolded titles for each of the figures within the body of the manuscript where they are first referenced. We did not include titles or captions in the .TIFF files as detailed in the PLOS One guidelines:

**“Place** figure captions in the manuscript text in read order, immediately following the paragraph where the figure is first cited. Do not include captions as part of the figure files or submit them in a separate document.”

Could not find figure 1.

Thank you. It seems that figure 1 did not properly upload into the PDF though it was submitted along with the other figures. We will ensure that figure 1 is appropriately uploaded with this submission.

References:

1. Please provide more details for Ref#5. If it is a website, please provide the URL and the date when it was accessed for the last time.

Thank you, we have provided the URL and date last accessed.

2. Please provide more details for Ref#14. If it is a report available on a website, please provide the URL and date when it was accessed for the last time.

Thank you, we have provided the URL and date last accessed.

3. Please correct the author's name of the Ref#16.

Thank you, we have corrected the author’s name.

4. Please correct the NFHS-4 reference (Ref#24). In the author list, Macro international should be replaced by ICF. Need to add the same in Ref#16 as well.

Thank you, we have corrected the author’s name.
